# New Drugs and Therapies in Pulmonary Arterial Hypertension

**DOI:** 10.3390/ijms24065850

**Published:** 2023-03-19

**Authors:** Aangi J. Shah, Taylor Beckmann, Mounica Vorla, Dinesh K. Kalra

**Affiliations:** 1 Department of Medicine, University of Louisville, Louisville, KY 40202, USA; 2 Division of Cardiology, Department of Medicine, University of Louisville, Louisville, KY 40202, USA

**Keywords:** pulmonary arterial hypertension, novel treatments, novel therapies, new drug targets, pathogenesis

## Abstract

Pulmonary arterial hypertension is a chronic, progressive disorder of the pulmonary vasculature with associated pulmonary and cardiac remodeling. PAH was a uniformly fatal disease until the late 1970s, but with the advent of targeted therapies, the life expectancy of patients with PAH has now considerably improved. Despite these advances, PAH inevitably remains a progressive disease with significant morbidity and mortality. Thus, there is still an unmet need for the development of new drugs and other interventional therapies for the treatment of PAH. One shortcoming of currently approved vasodilator therapies is that they do not target or reverse the underlying pathogenesis of the disease process itself. A large body of evidence has evolved in the past two decades clarifying the role of genetics, dysregulation of growth factors, inflammatory pathways, mitochondrial dysfunction, DNA damage, sex hormones, neurohormonal pathways, and iron deficiency in the pathogenesis of PAH. This review focuses on newer targets and drugs that modify these pathways as well as novel interventional therapies in PAH.

## 1. Introduction

Pulmonary arterial hypertension (PAH) is a chronic, progressive, pan vasculopathy affecting the pulmonary vasculature, with predominant pathology initiated in the pre-capillary pulmonary vascular tree, culminating in an elevated pulmonary vascular load [1]. Pulmonary hypertension (PH), in general, consists of a mixed group of disorders, all of which eventually lead to an elevation in pulmonary arterial pressure [2]. PAH is clinically classified by the 6th World Symposium on Pulmonary Hypertension (WSPH, 2018) as Group 1 Pulmonary Hypertension, based on the underlying etiology. Other clinical subgroups include Group 2 PH, which develops due to underlying heart failure (reduced or preserved ejection fraction), valvular heart disease, or congenital heart diseases. Group 3 PH arises due to lung diseases or hypoxia. Group 4 PH evolves due to pulmonary artery obstructions, which also includes chronic thromboembolic PH (CTEPH) [3]. CTEPH is characterized by chronic thrombi organization in the pulmonary arterioles with subsequent fibrosis and stenosis of the vessels [4]. Group 5 PH is a complex cohort often caused by multifactorial etiologies [3].

PAH, when defined hemodynamically, is classified as pre-capillary PH given that the underlying hemodynamic changes affect mainly the pre-capillary pulmonary vasculature [5]. The numeric threshold for defining PH was initially arbitrarily defined as per expert consensus at the 1st WSPH (1973) as mean pulmonary artery pressure (mPAP) ≥ 25 mm Hg. However, further studies by Kovacs et al. [6,7], analyses on scleroderma patients [1,8], and a large retrospective review by Maron et al. noting elevated all-cause mortality and hospitalization for mPAP ≥ 19 mmHg [9], provided evidence that mPAP threshold for disease causation was much lower than the previously defined 25 mm Hg. The 6th WSPH in 2018 redefined PH as mPAP ≥ 20 mm Hg, twice the upper limit of a normal mPAP of 14.0 ± 3.3 mm Hg as reported by Kovacs et al. in 2018 [7]. The 2022 European Society of Cardiology (ESC)/European Respiratory Society (ERS) guidelines have defined PH as mPAP > 20 mm Hg at rest, and pre-capillary PH in Group 1 PAH as both pulmonary vascular resistance (PVR) < 2 Wood units (WU) and pulmonary artery wedge pressure (PAWP) < 15 mm Hg [3].

## 2. Pathophysiology of Pulmonary Arterial Hypertension

The pathophysiology of PAH is complex and variable given the multiplicity of molecular mechanisms and the underlying disorders implicated in the pathogenesis. Intricate crosstalk among various signaling pathways involving nitric oxide, prostacyclin, serotonin along with neurohumoral and hormonal pathways, genetic predisposition with epigenetic modifications, mitochondria related metabolic milieu dysregulation, and environmental and inflammatory insults, all participate to form a complex endophenotype resulting in the pathological changes noted in PAH [10]. However, the most common pathological features, irrespective of the initial instigating injury, are pulmonary artery endothelial cell (PAEC) dysfunction, pulmonary artery smooth muscle cell (PASMC) proliferation and migration, and dysregulated fibroblast activity [11]. These lead to a phenotype of dysregulated vasoconstriction, micro and in-situ vascular thrombosis, vascular fibrosis, and pathogenic remodeling of pulmonary vessels. These processes have been mainly reported to affect the small and medium sized precapillary arterioles, which are 50–500 μm in diameter. Smooth muscle cell migration leads to muscularization of otherwise non-muscularized distal capillaries [12].

PAEC dysfunction has been widely reported as a key prong in PAH pathogenesis [11,13]. PAEC dysfunction causes an increased adhesive capacity, along with a proliferative and anti-apoptotic phenotype in the endothelium, thus increasing the burden of micro-thromboses and vessel wall hyperplasia [14,15]. The consequent smooth muscle dysfunction with excessive proliferation and migration leading to muscularization of arterioles further exacerbates the disease phenotype with ensuing luminal narrowing [11,16]. In addition, aberrant mitochondrial metabolic milieu secondary to aerobic glycolysis further promotes a pro-proliferative endophenotype. This Warburg effect, such as the one described in cancer, occurs due to upregulation of pyruvate dehydrogenase kinase (PDK) that phosphorylates and inactivates the pyruvate dehydrogenase (PDH) [17,18]. Calcium ion mishandling is seen in PAH and causes the subsequent downregulation of potassium Kv.1.5 channels and activation of the nuclear factor of activated T-cells (NFAT). The former furthers the vasoconstrictive phenotype, while the latter results in apoptosis resistance with upregulation of bcl-2 [17,19,20]. Dysregulated calcium handling has also been reported due to impaired calcium uptake via the mitochondrial calcium uniporter (MCU) and hyperactive RhoA/Rho kinase pathway [21,22].

A nitric oxide (NO) pathway has been widely reported as an important mediator of PAH pathogenesis. Decreased bioavailability of NO and decreased expression of endothelial nitric oxide synthase (eNOS)—or rather dysfunctional eNOS—results in an imbalance favoring vasoconstriction, dysregulation of smooth muscle cell proliferation, in-situ platelet thrombosis, and overproduction of collagen products. Vasoconstriction has also been reported in PAH secondary to the imbalance between thromboxane A2 (TXA_2_) and prostacyclin (PGI_2_), and the role of prostanoids is thus established in the treatment of PAH. On similar lines, endothelin (ET) dysfunction, secondary to inflammatory insults, hypoxia, and hormonal factors, also predisposes to a vasoconstrictive phenotype in the pulmonary vessels. These pathways form the basis of the currently approved therapies for PAH.

Neurohumoral pathways with increased activity of angiotensin converting enzyme (ACE), elevated aldosterone levels [10], and with the role of estrogen in the downregulation of the BMPR2 gene have also been reported in PAH pathogenesis [10,23]. Furthermore, an expanding body of evidence reports on the role of inflammation and elevated levels of circulating cytokines and chemokines (interleukin (IL)-1β, IL-6, IL-8, monocyte chemoattractant protein (MCP)-1, fractalkine, CCL5/RANTES, and tumor necrosis factor (TNF)-α). The pathology of PAH lesions show perivascular infiltrate comprising of T-cells, B-cells, macrophages, dendritic cells, and mast cells [13,16].

**Figure 1:**
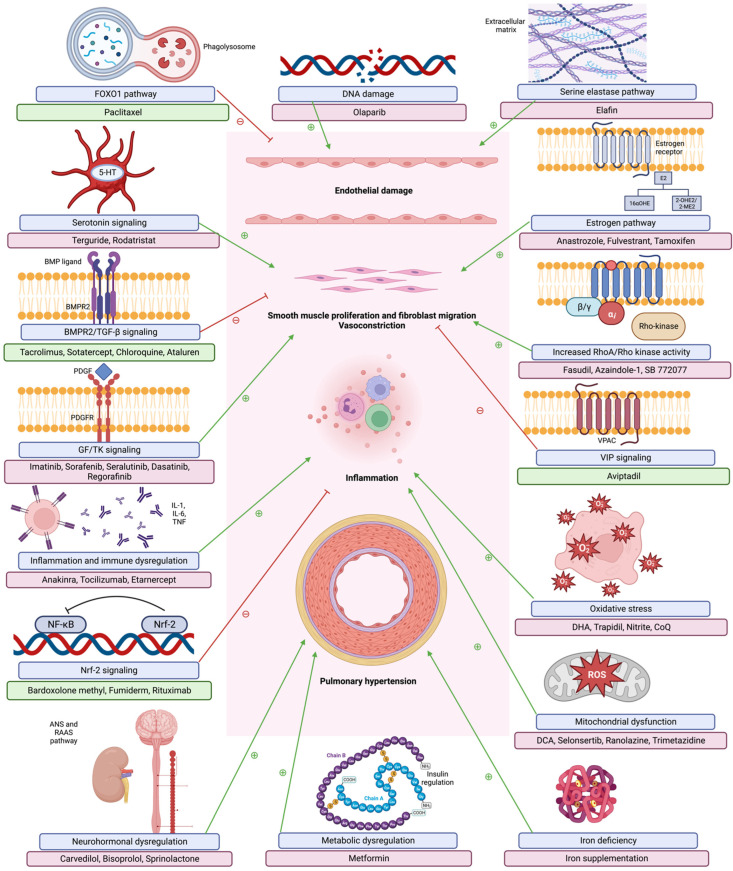
Pulmonary arterial hypertension (PAH) is the result of complex interplay between multiple pathways and environmental and genetic stressors. This figure is a pictorial representation of the multiple pathogenetic pathways observed in PAH, with the novel therapeutic options available to target the disease process. Green arrow = activator of the pathway, red arrow = inhibitor of the pathway, blue box = pathway, red box = inhibitor of the pathway, green box = activator/modulator of the pathway. 16αOHE = 16 Alpha Hydroxyestrone, 2-ME2 = 2 Methoxy estradiol, 2OH-E2 = 2-hydroxyestrone, 5-HT = 5-Hydroxy Tryptamine, ANS = Autonomic Nervous System, BMP = Bone Morphogenetic Protein, BMPR2 = Bone Morphogenetic Protein Receptor Type 2, CoQ = Coenzyme Q, DCA = Dichloroacetate, DHA = Dihyrodroatremisinin, DNA = Deoxyribonucleic Acid, GF = Growth Factor, IL = Interleukin, NF-κB = Nuclear Factor Kappa B, Nrf-2 = Nuclear Factor E2-related factor 2, PDGF = Platelet-Derived Growth Factor, PDGFR = Platelet-Derived Growth Factor Receptor, RAAS = Renin-Angiotensin-Aldosterone System, ROS = Reactive Oxygen Species, TGF β = Transforming Growth Factor beta, TK = Tyrosine Kinase, TNF = Tumor Necrosis Factor, VIP = Vasoactive Intestinal Peptide, VPAC = Vasoactive Intestinal Peptide Receptor.

These pathways show the development of occlusive arteriopathy, with elevated pulmonary vascular load and subsequently elevated right ventricular pressure overload. Maladaptive right ventricular (RV) dilation ensues, with right ventricular (RV)-pulmonary artery (PA) uncoupling and RV failure [24]. Figure 1 describes multiple pathogenetic pathways noted in PAH and their relevant therapeutic interventions.

## 3. Current FDA Approved Therapies for Pulmonary Arterial Hypertension

The latest ESC/ERS guidelines recommend a step-wise approach to the treatment of PAH, and triaging therapy decisions using initial analysis with World Heart Organization (WHO) functional staging [3,25]. Primary staging is recommended using the various risk stratification tools (Registry to Evaluate Early and Long-Term PAH Disease Management (REVEAL) risk scores, ESC/ERS risk-stratification table) that incorporate cardiopulmonary hemodynamic measurements. Diagnosis is established through a three-pronged approach with clinical suspicion, supportive imaging evidence (echocardiogram, cardiac MRI), and confirmation with right heart catheterization in specialized PH centers.

Patients with idiopathic PAH (iPAH), hereditary PAH (hPAH), or drug/toxin induced PAH are recommended to first undergo vasoreactivity testing (VT), which—if positive—merits an initial trial with calcium channel blockers (CCBs) [3]. However, less than ten percent of patients in these PAH subtypes respond positively to VT. Furthermore, an initial positive vasodilation response does not predict long term responsiveness CCBs. Less than five percent of the initially VT responsive patients sustain the response at one year [26]. There are no randomized controlled trials (RCT) evaluating the mortality in VT responsive patients treated with CCBs, and thus it has been a clinical uncertainty if CCB therapy ameliorates the PAH disease process or if the presence of vasoreactivity is in itself a positive prognostic marker [27].

Ten pharmaceuticals have been approved by the Food and Drug Administration (FDA) of the United States for the treatment of PAH. They can be grouped under five drug classes [28]. Endothelin-1 binds to endothelin receptors A and B (ET_A_, ET_B_) on PASMCs and induces both vasoconstriction via phospholipase C-Β and inositol triphosphate induced elevation in intracellular calcium. In addition, endothelin-1 has also been reported to induce fibrogenesis, which has been observed in PAH secondary to its action on matrix metalloproteinases and smooth muscle hypertrophy that occurs in concert with micro-environmental factors such as hypoxia, oxidative stress, inflammatory mediators, and hormonal agents [19]. **Ambrisentan**, **bosentan,** and **macitentan** are three endothelin receptor antagonists (ERAs) approved by the FDA [2]. Sitaxentan, previously approved, has been banned after reports of fulminant hepatotoxicity observed with it [29,30].

Nitric oxide acts on soluble guanylate cyclase (sGC) in the pulmonary vascular smooth muscle, and stimulates cyclic guanosine monophosphate (cGMP) production, that in a cascade of events, brings about vasodilatation [31]. cGMP is degraded by the phosphodiesterase (PDE) enzymes, with PDE5 being majorly expressed in the pulmonary vasculature. PDE5 inhibitors, namely **sildenafil** and **tadalafil**, exert their therapeutic effect via stimulating pulmonary vasodilation [32]. **Riociguat** is a sGC stimulator that acts independently of NO. These group of agents also increase sensitivity of sGC to NO, and the latter might be diminished or insufficiently produced in PAH [33,34].

As described previously, prostacyclin pathways are dysregulated in PAH with a decrease in levels of PGI_2_ and decreased excretion of prostacyclin urinary metabolites, with consequent elevated elevation of TXA_2_, and exacerbation of the vasoconstrictive phenotype in PAH [10]. Prostacyclin analogs approved by the FDA include **epoprostenol**, **iloprost**, **treprostinil**, **beraprost** and **selexipag**.

**Figure 2:**
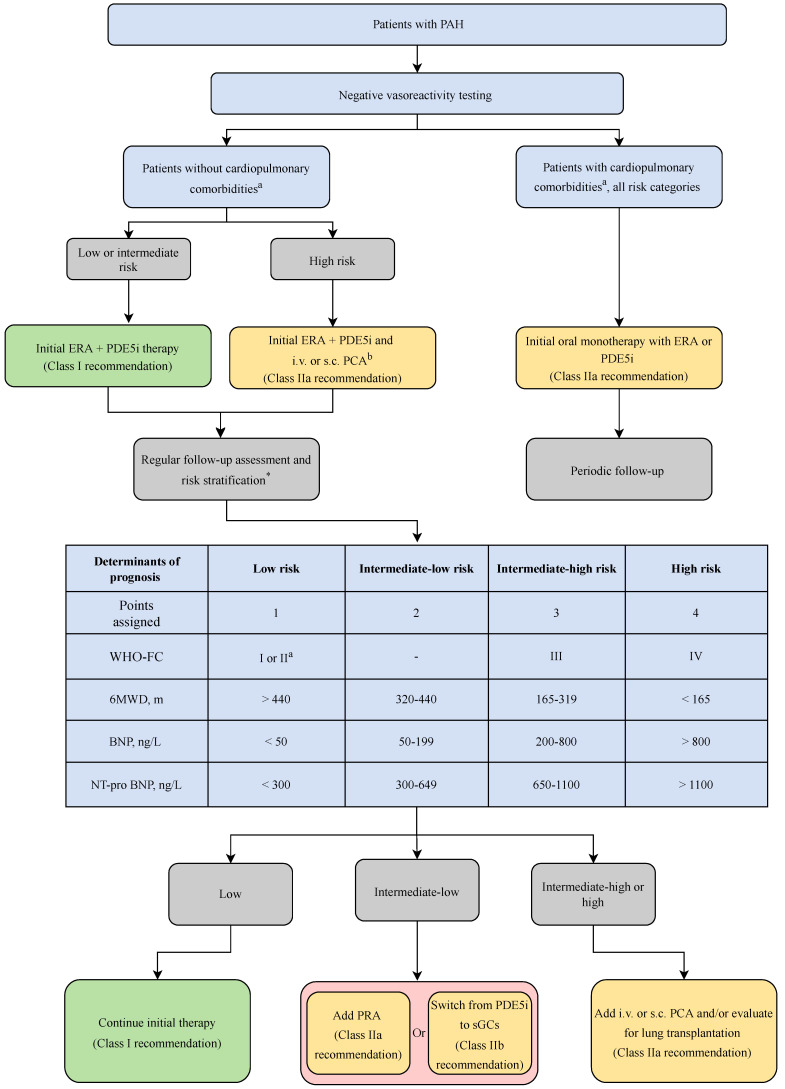
University of Louisville School of Medicine approach to pulmonary arterial hypertension treatment. PAH = Pulmonary Arterial Hypertension, 6MWD = 6Minute Walk Distance, BNP = Brain Natriuretic Peptide, DLCO = Lung Diffusion Capacity for Carbon Monoxide, ERA = Endothelin Receptor Antagonist, i.v. = Intravenous, NT–pro BNP = N–Terminal Pro–Brain Natriuretic Peptide, PCA = Prostacyclin Analogue, PDE5i = Phosphodiesterase 5 Inhibitor, PRA = Prostacyclin Receptor Agonist, s.c. = Subcutaneous, sGCs = Soluble Guanylate Cyclase stimulator, WHO–FC = World Health Organization Functional Class, ng = Nanogram, L = Liter. * Risk is calculated by dividing the sum of all grades by the number of variables and rounding to the next integer. ^a^ Cardiopulmonary comorbidities are conditions associated with an increased risk of left ventricular diastolic dysfunction, and include obesity, hypertension, diabetes mellitus, and coronary heart disease; pulmonary comorbidities may include signs of mild parenchymal lung disease and are often associated with a low DLCO (<45% of the predicted value). ^b^ Intravenous epoprostenol or i.v./s.c. Treprostinil.

Current guidelines recommend initiation of combination therapy in patients diagnosed with low to intermediate risk of death and not qualifying for VT [35]. Figure 2 describes the latest evidence-based guidelines for the treatment of PAH.

Until two decades back, treatment of PAH was based on best clinical decision due to a paucity of evidence, the majority of it based only on scattered case reports [28]. With better understanding into the pathophysiology of PAH and its underlying mechanisms, the last twenty years have seen the arrival of multiple PAH specific therapies. In more recent years, there have been significant changes in the designing of the clinical trials to provide more clinical relevance to the trial findings. Firstly, the RCTs now categorize end points in a more composite fashion (a combination of disease process worsening, hospitalizations, therapy escalation and mortality) instead of more discrete functional end points such as improvement in 6 min walk distance (6MWD) test, as this purports higher clinical relevance and generalizability to the study outcomes. Furthermore, RCTs are increasingly evaluating the action of combination therapy or add-on therapy to established background treatment [12]. Prior to the approval of epoprostenol for treatment of PAH in 1995, no disease specific targeted therapy existed [36]. However, despite this ascent and evolution of research into the pathology and clinical treatment of PAH, the survival data still shows poor prognoses, and PAH continues to remain a fatal disease [37]. An important reflection about current PAH therapies is that they focus predominantly on the vasodilation of partially occluded vessels, despite reports that less than ten percent of patients afflicted with PAH have a dominant vasoconstrictive endophenotype [17]. In addition, with the recognition of BMPR2 variants in PAH pathogenesis, it was noted that less than thirty percent of these patients have single causative gene variations, thus indicating the role of multiple micro-environmental, inflammatory, hormonal, metabolic and post-translational mechanisms in the causation of PAH [38]. These speak to a pressing need for disease-modifying therapies targeting relevant pathophysiological pathways for PAH.

## 4. Novel Therapies

### 4.1. BMPR2 Pathway

Genetic pathways are known to play a large role in the pathogenesis of PAH and the bone morphogenetic protein receptor 2 (BMPR2) gene is well established as the most common site of mutations implicated in PAH. Although the REVEAL registry lists the incidence of heritable PAH at less than 5% of known cases, mutations in the BMPR2 gene (a member of the transforming growth factor-β superfamily) have been identified in up to 40% of non-heritable PAH cases along with 80% of heritable cases [3,39]. PAH patients with a BMPR2 mutation are also known to present at a younger age with a more severe phenotype and an increased risk of death [40]. Mutations at this gene carry variable penetrance, approximately 27%, but this number differs by sex with female occurrences predominant (40%) to male occurrences (14%) [17]. The presence of a BMPR2 mutation increases an individual’s chance of developing PAH from approximately 1 in 100,000 to 1 in 4 [17]. However, these mutations still require further inciting factors to permit disease, such as epigenetics, acquired mutations, or environmental factors.

Back in 2000, with the advent of rapid and financially feasible genome sequencing technology, the BMPR2 gene was discovered to be involved in the pathogenesis of familial PAH, with germline mutations noted throughout families with a strong inheritance pattern of PAH [39]. Since then, more than 300 different *BMPR2* mutations have been identified. Genetic sequencing of the BMPR2 receptor and its associated downstream signaling cascade have revealed sequence variants related to BMPR2 signaling in ALK1, CAV1, ENG, SMAD4, SMAD8, SMAD9, BMPR1, and BMP9 genes [41]. A large study cohort found CAV1 mutations in *BMPR2*-negative individuals with more than one family member diagnosed with PAH. Caveolin-1 functions to physically colocalize BMP receptors [42].

The BMPR2 receptor is a serotonin/threonine kinase receptor in the transforming growth factor-B (TGF-β) superfamily that activates different signaling cascades. Many of these cascades occur through either SMAD 2/3 (TGF-β) or SMAD 1/5/8 (BMP) [43]. Mutations at the BMP receptor cause loss-of-function and dysregulated downstream signaling of this gene. Though the BMPR2 gene can be found many places in the body, much of its expression is found in the pulmonary vasculature in PAECs and PASMCs. Loss of signaling here results in greater TGF-β activity, promoting both proliferative and anti-apoptotic responses in PAECs and PASMCs with increased inflammatory cytokine production. This further cascades into loss of regulated cellular apoptosis, maladaptive vascular remodeling, and endothelial inflammation [43]. Dewachter et al. showed a lower mRNA expression of BMPR2 in isolated PASMCs in both hPAH and iPAH patients [44]. These findings place BMPR2 expression at the center of the pathophysiology of PAH and makes it a critical target for therapy.

#### 4.1.1. BMP Ligands

BMP receptors require ligand binding for activation of their intracellular MAP-kinase functions, given that therapeutic targets of PAH are geared towards restoration of BMPR2 function.

Once such compound is **FK506**, or **tacrolimus**, an FDA-approved calcineurin inhibitor used for postoperative immunosuppression and management of autoimmune diseases. Tacrolimus indirectly activates BMPR2 by removing an inhibitory binding protein from BMPR1 that restores normal MAP-kinase function to this complex. A separate mechanism of the action of the drug works by inhibiting a phosphatase that phosphorylates both the type 1 receptors and downstream SMAD signaling [45]. Mouse models with tacrolimus have shown reversal of RV fibrosis and endothelial dysfunction. A single center, phase IIa randomized controlled trial (TransformPAH) with 23 patients showed enhanced BMPR2 expression after tacrolimus administration [46]. This study investigated tacrolimus to achieve three separate serum levels over 16 weeks. Tacrolimus was found to be safe and well tolerated in patients on current-guideline-directed PAH therapy, but it was not powered to evaluate for efficacy [46]. More trials are needed to examine the efficacy of tacrolimus.

Another molecule acts on BMPR2 signaling through its balance with TGF-β. TGF-β activity is known to be increased in the pulmonary vasculature in mouse models of PH [47]. Increased TGF-B signaling decreases BMP signaling and vice versa, thus imbalancing and over-promoting TGF-β, leading to endothelial dysfunction and inflammation [48]. **Sotatercept** (ActRIIa-Fc) utilizes a TGF-β ligand trap to inhibit TGF-β activity and rebalance BMPR2 activity. The phase II PULSAR trial reduced PVR and improved 6MWD, N-Terminal pro-Brain Natriuretic Peptide (NT-proBNP), and WHO functional class [28,45]. Phase III trials (STELLAR) are being conducted in FC-III patients along with more phase II (SPECTRA) trials investigating RV function and exercise tolerance, using cardiopulmonary rehab and cardiac MRI to evaluate these parameters [45].

#### 4.1.2. Other BMPR2 Modulating Therapies

There are many other investigational drugs that target various mechanisms to increase BMPR2 signaling. Chloroquine inhibited the development and progression of pulmonary hypertension in monocrotaline-exposed rats [49]. Monocrotaline (MCT) is an alkaloid derivative that can cause PH [50]. **Chloroquine** has the proposed effect of preserving lysosome-targeted degradation of BMPR2 by blocking the last step in the autophagy pathway. Rat models confirmed that chloroquine both inhibited proliferation and increased apoptosis of PASMCs, assumed to be from increased BMPR2 expression [49].

**Ataluren** (**PTC124**) is a small molecule that is under investigation for the treatment of multiple inherited disorders such as Duchenne muscular dystrophy, cystic fibrosis, and PAH. Ataluren are proposed to interact with ribosomes and suppress nonsense mutations through tRNA activity to allow synthesis of full-length proteins without affecting normal translational stop signals [51]. In vitro studies of explanted lungs from hPAH patients showed increasing BMPR2 protein levels and increased SMAD phosphorylation [51,52]. Clinical trials investigating Ataluren in PAH are needed.

### 4.2. Inflammation and Immunity

In recent years, ample literature has reported on the role of inflammation and dysregulated immune response in the causation of PAH. In fact, authors have likened PAH to an autoimmune disease itself [53]. PAH is frequently observed in patients with systemic autoimmune diseases, notably, systemic sclerosis, systemic lupus erythematosus, and mixed connective tissue disease. Right heart catheterization data reports the presence of elevated mPAP, establishing the diagnosis of PAH in 8–12% of patients with systemic sclerosis [54]. On the flip side, a significant proportion of patients with idiopathic PAH report the presence of Raynaud’s phenomenon. Similarly, the pulmonary vascular pathology—namely the plexiform lesions—observed in autoimmune diseases and viral infections are akin to the ones observed in idiopathic PAH, suggesting comparable if not consistent underlying mechanisms affecting both the etiologies of PAH [55]. These pathological lesions seen in PAH have been observed to have variable degrees of perivascular inflammatory infiltrate comprising of T-cells, B-cells, macrophages, dendritic cells, and mast cells. In addition, recent studies on a scoring system analyzing the relationship between mPAP and average perivascular inflammation and pulmonary vessel wall thickness report a positive correlation between pulmonary vascular remodeling and perivascular inflammation [56].

Elevated levels of circulating cytokines and chemokines, such as interleukin (IL) -1β (IL-1β), IL-6, and tumor necrosis factor (TNF)-α have been measured in patients with PAH [11]. Furthermore, higher levels of such inflammatory mediators have been associated with poorer prognoses [57,58]. This might be secondary to the observation that higher levels of chemokines and cytokines exaggerate vasoconstriction, vascular proliferation, and fibrosis, exacerbating the vascular remodeling [16]. Various autoimmune antibodies such anti-nuclear, anti-endothelial cells, and anti-fibroblast antibodies are seen in iPAH and systemic sclerosis [59,60]. The T-regulatory cells (Treg) have been long associated with maintenance of self-tolerance and protection against autoimmunity [16]. The absence of Treg cells has been noted in patients with PAH [61]. Athymic hypoxia model rats were noted to develop perivascular inflammation and vascular remodeling with severe PAH phenotypes, which was otherwise averted by Treg cells’ reconstitution [62,63].

Several clinical studies are currently evaluating the role of immunoregulatory agents in the treatment of PAH.

#### 4.2.1. Interleukin-6 (IL-6)

Elevated IL-6 has been implicated in the cytokines involved in the pathogenesis of PAH. Inept p38 signaling due to loss of BMPR2 in pulmonary vascular cells results in augmented IL-6 production. Heightened IL-6 levels in preclinical studies have been associated with PAH. Rodent models lacking IL-6 have shown decreased inflammation and pulmonary vascular phenotype of PAH, while IL-6 antagonists have abated the development of pulmonary vascular remodeling and PH (PAH is still called PH in animal models). Human studies have reported elevated IL-6 levels in pulmonary vasculature of patients with PAH [11].

**Tocilizumab** is a monoclonal antibody against the IL-6 receptor, and it is used to treat systemic inflammatory conditions such as rheumatoid arthritis, systemic sclerosis, giant cell arteritis, and cytokine release syndrome. Anecdotal data from case reports have reported clinical improvements in patients with PAH treated with the anti-IL-6 antibody [64,65,66]. Given this evidence, TRANSFORM-UK (Therapeutic Open-Label Study of Tocilizumab in the Treatment of Pulmonary Arterial Hypertension, NCT02676947) was conducted as a phase II open label clinical trial evaluating the safety and efficacy of tocilizumab (8 mg/kg) over 6 months in 29 patients (23 patients received study drug) with PAH. In addition, a separate mendelian randomization study was also conducted on 11,744 patients of European ancestry regarding the occurrence of the IL-6 receptor (IL-6R) variant (rs7529229). The primary outcomes were safety and change in pulmonary vascular resistance. The study, however, failed to show any consistent treatment effect, and adverse events observed were consistent with the hitherto known side effect profile of tocilizumab. In the study, the inflammatory markers (IL-6 and C-reactive protein (CRP)) were not predictive of the treatment response, as despite a positive trend in both the markers, there was no significant change observed in the PVR. Furthermore, mendelian randomization did not show any association of the IL-6R variant with the risk for developing PAH [67].

#### 4.2.2. Interleukin-1 (IL-1)

Elevated levels of IL-1 have been noted in PAH. Osteoprotegerin (OPG) was noted to be elevated in human PAH lesions. It was also observed that recombinant OPG stimulation resulted in PASMC proliferation and migration in vitro. OPG protein is closely regulated by BMP, IL-1, and serotonin signaling [68]. Voelkel et al. reported in the 1990s on elevated levels of IL-1 mRNA in the lungs of monocrotaline (MCT) treated rats, with ‘ubiquitous staining’ noted in alveolar structures, pulmonary vascular cells, and bronchial smooth muscle cells. In addition, the authors also accounted for improvement in mPAP with IL-1 receptor (IL-1R) antagonist and reduction in RV hypertrophy in MCT models. This effect was not true for hypoxia rodent models [69,70].

**Anakinra** is an interleukin-1 receptor (IL-1R) antagonist used in the treatment of rheumatoid arthritis. Case reports have described an improvement in PAH associated with other inflammatory conditions when treated with anakinra [71]. Trankle et al. devised a single group open label phase IB/II pilot study including six patients with group 1 PAH evaluating the role of anakinra as an add-on therapy to a hitherto established standard of care therapy for PAH. The patients were noted to have significant improvements in heart failure symptoms as assessed by the Minnesota Living with Heart Failure Questionnaire and improvement in disease severity as assessed with Duke Activity Severity Index scores. Study patients also reported a reduction in high-sensitivity CRP as compared to the baseline. However, no significant changes were reported in exercise time, NT-pro BNP, change in RV fractional area, or tricuspid annular plane systolic excursion [72].

#### 4.2.3. Tumor Necrosis Factor-α (ΤΝF-α)

Overexpression of TNFα has been reported in PAH animal models and in human subjects [73,74]. TNFα has been noted to lead to increased pulmonary vascular reactivity by attenuating prostacyclin production in PASMC. TNFα stimulates PASMC proliferation via the platelet derived growth factor (PDGF) pathway [75,76]. Studies have also reported an improvement in pulmonary vascular resistance by suppressing TNF production with high doses of pentoxifylline [77]. TNFα mediated subverted signaling of BMP2 pathways, and has been described to be responsible for PASMC proliferation. TNFα is also responsible for increasing pro-proliferative NOTCH-2 gene signaling via Src kinases in PASMC with impaired BMPR2 gene expression [78].

**Etanercept** is a biologic fusion protein that is a TNFα inhibitor. It is used, akin to the previously described biologics, to treat systemic inflammatory conditions such as rheumatoid arthritis, psoriatic arthritis, juvenile idiopathic arthritis, and ankylosing spondylitis. Etanercept has shown a deterrence of pulmonary hypertension in endotoxemic pig models [79]. Etanercept has also been observed to prevent and revert monocrotaline-induced pulmonary hypertension in rats. Immunohistochemistry staining reported exaggerated IL-6 and TNFα in model rats and decreased expression of the inflammatory cytokines in the etanercept treatment groups [80]. Currently, no active clinical trial is under effect evaluating the efficacy and safety of etanercept in PAH, but results from animal studies are promising and might pave the path to a clinical trial in the future.

#### 4.2.4. Nuclear Factor κβ

NF-κβ has been implicated as the underlying regulator of various immune pathways [11,81]. Studies on monocrotaline rat models have shown that nuclear localization of the p-65 subunit of NF-κβ with downstream vascular cell adhesion molecule (VCAM)-1 expression was temporally and spatially associated with the development of PH in these animals [82]. Similarly, human studies showed increased nuclear p65+ in macrophages of iPAH patients as compared to macrophages in control patients. Additionally, NF-κβ activation was increased in PAEC and PASMC in iPAH patients [83].

**Bardoxolone methyl** is a Nuclear factor E2-related factor 2 (Nrf2) activator and suppresses NF-κβ in human cell lines [84]. LARIAT (NCT02036970) was a phase-II randomized controlled trial recruiting 22 patients with PH associated with idiopathic pulmonary fibrosis, connective tissue disease associated interstitial lung disease, or non-specific interstitial pneumonia or Group III or V PH. Patients on bardoxolone methyl showed significant improvement in 6MWD at 16 weeks [85]. CATALYST (NCT02657356) was a double-blind, randomized, placebo-controlled trial that studied the safety and efficacy of bardoxolone methyl in patients with PAH related to connective tissue disease, however, the study was terminated prematurely due to the COVID-19 pandemic. Similarly, long term extension study RANGER (NCT03068130) evaluating long term effects in patients with PAH who had previously participated in clinical trials with bardoxolone methyl too was terminated prematurely in the light of exposure risk to these patients due to the COVID-19 pandemic.

**Fumaderm** was an oral formulation used in Germany to treat psoriasis. **Dimethyl fumarate** (**DMF**), which was approved in the US, is a reformulation of fumaderm. DMF is well-known for its effect on Nrf2, activating the transcription of detoxification enzymes and curbing oxidative damage. In addition, DMF also suppresses TNFα and IL-6 by decreasing the oxidative stress. In this regard, DMF is also acts as an immune modulator. Studies on the role of DMF in the treatment of PH using murine models have shown effectiveness in reversing the pulmonary vascular remodeling and hemodynamic changes in these animal models. The mechanism was postulated to be via inhibition of NF-κβ, STAT3, cJUN signaling, and degradation of pro-fibrogenic mediators such as β-catenin [86]. An investigator-initiated, double-blind, randomized, placebo-controlled clinical trial evaluating the role of DMF in PAH due to systemic sclerosis (NCT02981082) was terminated due to low recruitment. The results posted report recruitment of 6 patients, randomized to either DMF or placebo group. One participant in each group completed the protocol. The subjects in the DMF group showed a non-significant reduction in decline in the 6MWD test, however the study was not effectively powered to assess for efficiency. Thus, Nrf2 remains a therapeutic target that needs further studies assessing its role in the treatment of PAH [87].

**Rituximab**, is a monoclonal antibody against the CD20 receptor, targeting the B-cells of the immune system. Several incidental reports mentioned improvement of PAH with rituximab. Following this, an NIH-funded multicenter, double-blinded, randomized, placebo-controlled, proof-of-concept trial (NCT010865400) recruited 57 patients with systemic-sclerosis-related PAH on standard-of-care medical therapy, and were randomized to receive two infusions of rituximab (1000 mg) versus placebo, two weeks apart. The patients in the rituximab group showed an improvement in 6MWD at the 24 weeks follow up, but the data did not reach significance. While it was a negative study, evaluation of data collected through week 48 showed improvement in 6MWD at week 24 (rituximab 25.5 ± 8.8 m and placebo 0.4 ± 7.4 m, p 0.03). Rheumatoid factor, IL-12 and IL-17, were favorable predictors of response to the therapy. Further trials are required to corroborate the therapeutic response to B-cell depletion therapy [88].

### 4.3. GF/TK Signaling Pathway

Pulmonary vascular remodeling has now been described through various mechanisms in PAH; Not only proliferation of PAECs and PASMCs, but also abnormal increases in the production of growth factors such as vascular endothelial growth factor (VEGF) and PDGF. These changes ultimately lead to blood vessel narrowing, increased PVR, and increased pulmonary pressures [28]. The increased production of these growth factors, specifically PDGF, has researchers thinking of PAH as a neoplastic process. PDGF receptor activation stimulates fibroblasts and PASMCs proliferation. Previous animal models highlight that inhibiting the PDGF pathway may prevent or reverse the maladaptive proliferation of the intimal lining of the pulmonary arteries found in PAH [11]. PDGF and other growth factors exert their effect by binding to transmembrane tyrosine kinase receptors (TKRs) to activate tyrosine kinases (TKs). These enzymes trigger phosphorylation of tyrosine residues of specific proteins to either activate or inhibit several cellular functions, including the vascular remodeling in PAH [28]. Since therapies already exist to target these enzymes, some have been repurposed to explore their potential to treat PAH.

**Imatinib** is an oral chemotherapy agent used to treat various cancers such as chronic myeloid leukemia and gastrointestestinal stromal tumors. Imatinib inhibits several tyrosine kinase enzymes and was shown to reverse PDGF induced arterial remodeling in mice models [11,45]. Imatinib was the first TK inhibitor used in PAH clinical trials and showed promising results in the IMPRES trial, a phase III RCT on PAH patients currently on guideline recommended treatment. Results showed improvement in 6MWD and PVR. Unfortunately, high rates of serious adverse events, including subdural hematomas in patients on anticoagulation have halted further studies with oral imatinib [28,37,89]. An inhaled form of the drug was also developed to reduce systemic exposure. It is now being investigated in a phase II/III trial (IMPAHCT) (NCT05036135). AV-101, a dry powdered inhaler form of imatinib, will be investigated for safety and efficacy along with change in PVR over 24 weeks in this trial. The FDA granted AV-101 an orphan drug status in 2021.

The success of imatinib proved that tyrosine kinase inhibitors (TKI) have promise as a PAH treatment, but a more favorable profile was needed. **Seralutinib** was then developed specifically for PAH treatment. Seralutinib is a selective TKI that not only targets the PDGF cascade, but also increases BMPR2 expression. The dry powdered inhaler has shown reversal of PAH vascular remodeling and significant improvement in right ventricular systolic pressure in animal studies [90]. Seralutinib is now enrolling a phase II RCT trial (TORREY) evaluating its safety and efficacy in PAH FC-1 patients [90] (NCT04456998).

Other TKIs have been tested in PAH with high hopes, but none have had more favorable profiles [37]. **Sorafenib** is a multikinase inhibitor approved for treatment of renal cell carcinoma, acute myeloid leukemia, and other cancers [11]. Sorafenib has been repurposed due to its ability to inhibit angiogenesis of PDGF and VEGF receptors in rat models [91]. The rat models also showed improvement in cardiac and pulmonary functions [91]. A 16-week Phase Ib study showed PAH patients had a significant increase in their 6MWD with Sorafenib treatment but also demonstrated a decrease in their cardiac output, raising questions about the drug’s safety [92]. Significant side effects have been reported with sorafenib administration other than cardiac adverse events common to most TKIs, including serious skin reactions [92]. **Nilotinib** is another oral TKI used for gastrointestinal stromal tumors that blocks signaling specifically in the BCR-ABL signal transduction system [28]. The potency of nilotinib is 20–50 times greater than imatinib, and a phase II study to establish safety with this drug was terminated due to serious adverse events [11,28]. **Dasatinib**, a PDGF receptor inhibitor, was the only member of the TKI family to have a negative effect on PAH treatment [11,93]. This drug has been reported to induce PAH in multiple cases [28]. **Regorafenib**, an oral multi-targeted TKI, also showed promise in mouse models, but went on to show life-threatening cardiotoxicity in multiple case reports in humans [92]. Approximately 25% of patients treated with this drug had to discontinue due to cardiotoxicity [94]. The search for a viable TKI in PAH treatment without serious adverse reactions continues.

### 4.4. RhoA/Rho Kinase Pathway

RhoA is a guanosine-5′-triphosphate (GTP) binding protein that brings about its action via the Rho kinase pathway. RhoA-GTP complex stimulates phosphatase action of PTEN, a human tumor suppressor protein, which then prevents inappropriate cell mitogenesis and proliferation. Rho kinase participates in the calcium sensitization and homeostasis, and subsequently plays a role in the pathogenesis of PAH via impaired calcium regulation and PASMC proliferation [21]. Rho associated protein kinase (ROCK) also plays roles in cell cycle signaling, insulin signaling pathway, and regulation of membrane blebbing in apoptosis [95,96,97,98,99].

In vitro studies have noted increased rho kinase activity in circulating neutrophils in human PAH subjects as compared to healthy controls (*p* < 0.0001), and this rho kinase activity was also positively correlated with PAH severity and duration in these patients. In addition, the rho kinase pathway has also been implicated in the vasoconstrictive phenotype of PAH, with observations that endothelium-driven vasodilation diminished while serotonin-induced vasoconstriction (in the absence of endothelium) was accentuated in these patients. Researchers also reported improvement in serotonin-induced vasoconstriction with hydroxyfasudil, which is a rho kinase inhibitor [22]. 5-hydroxy tryptamine (5-HT) or serotonin mediated PASMC proliferation and platelet activation also converges via the rho kinase pathway [100].

**Fasudil**, also known as HA1077 or AT877, is a nonselective ROCK inhibitor. It is metabolized hepatically and converted into the active metabolite hydroxyfasudil [101]. It has been clinically used to prevent vasospasm after a subarachnoid hemorrhage [102]. In addition to fasudil, three other ROCK inhibitors have been evaluated in the treatment of PH. **Y**-**27632** was noted to result in vasodilation in hypoxic rat models with PH [103]. **Azaindole**-**1** was observed therapeutically beneficial in MCT, and hypoxia induced PH animal subjects, with improvement in right ventricular hypertrophy and pulmonary vascular hemodynamics [104]. **SB**-**772077** showed significant decrease in mPAP in MCT-induced PH in animal models [105].

Multiple trials have shown the short-term efficacy of intravenous and inhaled fasudil in in patients with PAH. Fukomoto et al. studied nine patients with severe PH (undiagnosed etiology) already on standard of care therapy to trial the acute effects of intravenous fasudil on pulmonary circulation. While effects on mPAP and cardiac index did not reach significance, all nine patients showed a 17% reduction in PVR (*p* < 0.05) [106]. Ishikura et al. studied the effects of intravenous fasudil injected over 30 min in eight female patients with PAH. Authors observed significant reductions in total pulmonary resistance and mPAP, with the lowest total pulmonary resistance noted from 30–60 min of drug administration [107]. Fujita et al. studied the efficacy of inhaled fasudil and inhaled nitric oxide in two protocols in fifteen patients with PAH. They observed that fasudil and nitric oxide both decreased mPAP (*p* < 0.01), but positive correlation with serum levels for high sensitivity CRP was noted only for fasudil [108]. Finally, Jiang et al. randomized 50 patients in a cross-over design to receive iloprost inhalation and intravenous fasudil. A comparable improvement in mPAP and PVR was observed with both the medications with fasudil, resulting in greater improvement in mean cardiac output, and mixed venous oxygen saturation [109].

Fukomoto et al. studied the mid-term effects of oral extended-release formulation of fasudil hydrochloride in 23 patients with PAH. This was a phase IIa double-blind, placebo-controlled pilot efficacy clinical trial. Patients were randomized per their 6MWD and received either fasudil or placebo in doses varying from two to six capsules daily for three months. While improvement in pulmonary hemodynamics and 6MWD did not reach significance, likely due to the inadequate power of the study, the improvement in cardiac index for the fasudil cohort as compared to placebo reached significance. The serum levels of hydroxyfasudil positively correlated with improvement in cardiac index in these patients [110].

### 4.5. Mitochondrial Dysfunction

Mitochondrial dysfunction is increasingly purported as a causative factor in PAH pathogenesis. Mitochondria are responsible for cellular redox reaction, oxidative glycolysis, regulation of cellular proton gradient and calcium homeostasis. Mitochondrial DNA lacks histone and thus has a higher susceptibility to oxidative damage [111]. Dysfunctional mitochondrial metabolism results in ‘aerobic’ glycolysis. PAEC, PASMC and even ventricular cardiomyocytes in PAH depart from normal coupled oxidative metabolism to the said aerobic glycolysis. This is also known as the ‘Warburg Effect’ and is a result of upregulation of pyruvate dehydrogenase kinase, upregulation of glucose transporters on cell membrane, increased activity of a variant pyruvate kinase, and mitochondrial hyperpolarization. Epigenetic silencing of the superoxide dismutase (SOD)-2 gene due to DNA methylation impairs antioxidant pathways in cells and activates hypoxia inducing factor (HIF)-1α, which is also incriminated in the said mitochondrial dysfunction [10]. This normoxic upregulation of HIF-1α is also responsible for neoangiogenesis and vasomotor dysregulation.

**Dichloroacetate** (**DCA**) is a pyruvate dehydrogenase kinase (PDK) inhibitor that is observed to reverse the glycolytic shift in mitochondrial glucose metabolism. By inhibiting PDK, DCA activates pyruvate dehydrogenase. In rodent studies, DCA has been shown to ameliorate the PH phenotype [112,113]. In MCT rats, DCA at therapeutic levels decreased RV fibrosis and hypertrophy [114]. A phase-I, two-center study recruited 30 patients with PAH functional class III-IV who were already established on standard of care therapy for 8 weeks prior to recruitment. The study was completed, but no results are available online (NCT01083524). Another 4-month, open-label study administered DCA to patients with iPAH already established on standard of care treatment, and they showed improvement in their mPAP, PVR and functional status, however the individual responses varied widely [115].

**Selonsertib** is an apoptosis signaling kinase (ASK)1 inhibitor. Mitogen activated protein (MAP) kinases—ASKI being part of this family—are activated by oxidative stresses. MAP kinases are implicated in the pathogenesis of PAH, with cell inflammation, proliferation, migration, and fibrosis. Animal studies on evaluating the role of ASK1 inhibitor (GS-444217) in MCT and Sugen/hypoxia rat models have shown dose-dependent reduction in mPAP and RV hypertrophy. In cellular models, the ASK1 inhibitor demonstrated reduced phosphorylation of p38 and c-Jun N-terminal kinase (JNK), with a subsequent reduction in the activity of primary mouse cardiac fibroblasts and pulmonary adventitial fibroblasts [116,117]. ARROW was a randomized, double-blind, placebo-controlled, phase-2, multicenter clinical trial evaluating the role of selonsertib in PAH patients, stratified by the PAH etiology (NCT02234141). Patients were randomly assigned to selonsertib 2 mg, 6 mg, 18 mg, or placebo administered orally once daily. At 24 weeks, the study participants did not demonstrate significant improvement in PVR. However, authors report that further studies should be devised evaluating the ASK1-p38 pathway for PAH patients [118].

**Ranolazine** is a piperazine derivative, initially approved by FDA in 2006 for treatment of chronic angina. It acts by inhibiting the late inward sodium current (I_NaL_) in the cardiac myocardium. I_NaL_ stimulates influx of sodium ions which promotes intracellular accumulation of Ca^+^ [2]. Intracellular calcium load begets further calcium release from intracellular sarcoplasmic reticulum. It also inhibits fatty acid oxidation at higher plasma concentrations. Inhibition of I_NaL_ and fatty acid oxidation are the probable mechanisms through which ranolazine inhibits pathogenic RV hypertrophy, thus preventing the disease phenotype [119]. MCT-induced PAH rat models treated with ranolazine demonstrated decreased intracellular calcium overload and improvement in RV hypertrophy, RV pressure, and plasma BNP levels [120,121]. Ranolazine has also been shown to ameliorate doxorubicin-induced cardiotoxicity in experimental models via combating oxidant stress [122].

In a single-center, phase I, placebo-controlled, randomized clinical trial designed to assess vasoreactivity and safety of ranolazine in PAH, researchers enrolled 12 patients to receive either placebo or ranolazine (NCT01757808). Only one study group patient and two additional patients during open-label trial had ranolazine plasma concentrations reaching therapeutic concentrations. At 12 weeks, the study participants noted no significant improvement in hemodynamics as compared to the placebo group. However future studies would be needed to determine PK analysis in patients on other PAH therapies to consider the safety and tolerability of high dose ranolazine needed to achieve a therapeutic effect [123]. Khan et al. designed an open label, 3-month, pilot study recruiting 11 patients with symptomatic PAH and echocardiographic evidence of RV dysfunction to receive ranolazine (NCT01174173). While invasive hemodynamic monitoring parameters did not show significant improvement, researchers observed improvement in symptoms and echocardiographic parameters [124]. Han et al. devised a longitudinal, randomized, double-blind, placebo-controlled, multicenter clinical study in 24 patients with PAH and RV dysfunction (RV ejection fraction < 45%). The study has been completed, but the results are yet to be reported [125].

**Trimetazidine** is another piperazine derivative used to treat angina. It has been postulated that trimetazidine inhibits fatty acid oxidation and helps ameliorate the PAH phenotype. The literature reports that trimetazidine inhibits malonyl coenzyme A decarboxylase (MCD), thereby normalizing mitochondrial oxidative metabolism and alleviating the PAH phenotype [126]. Researchers in Chile are currently recruiting patients for a randomized, cross-over clinical trial evaluating the effect of trimetazidine on RV function, remodeling and miR profiling in PAH (NCT012102672).

### 4.6. Oxidative Stress

Oxidative stress is an inherent imbalance between reactive oxygen species (ROS), reactive nitrogen species (RNS), and free radicals and antioxidant pathways. Oxidative species comprise of singlet oxygen, hydroxyl radical, hydrogen peroxide, superoxide anion, nitric oxide, and peroxynitrite. DNA damage, because of oxidative stress, is observed to be increased in PAEC and PASMC in human PAH patients. Oxidative stress is associated with BMPR2 downregulation in human PAECs, and this results in downregulation of downstream DNA repair genes such as ataxia telangiectasia mutated (ATM) and breast and ovarian cancer susceptibility protein 1 (BRCA2) [111,127]. PAH susceptibility is also elevated with mutations in DNA topoisomerase binding protein, which results in unregulated PAEC and PASMC proliferation, and apoptosis inhibition, contributing to the malignant phenotype often described in PAH [128].

ROS and RNS causing oxidative insults trigger increased expression and activation of growth factors such as platelet derived growth factor (PDGF), p38-mitogen activated protein kinase (MAPK), VEGF, TGF-β, and fibroblast growth factor (FGF) [129]. Activation of TGF-β pathway elicits endothelial to mesenchymal proliferation of PASMC [130]. PDGF is culpable for promoting PASMC proliferation and migration, along with apoptosis resistance in PAEC [131]. The endothelial plexiform lesions are also a result of PDGF stimulation by oxidative mechanisms [132].

Given this wealth of data, numerous pre-clinical studies have been devised to evaluate the role of antioxidants in the treatment of PAH. Table 1 enumerates the multiple preclinical animal studies with antioxidants and showing effect with improvement of PAH [129].

**Dihyrodroatremisinin** (**DHA**) has been evaluated as an *anti*-*hypoxia* agent in experimental animal models such as MCT rats [133]. DHA diminished hypoxia-induced PAEC proliferation and migration in a hypoxic human PAEC model. The mechanism was understood to be via reduction in ROS and RNS and increase in SOD [134]. While otherwise an anti-malarial agent, DHA also modulates its effect in PAH treatment via microRNA (miR) regulation. Studies in in vitro and in vivo settings have shown that DHA limits hypoxia induced upregulation of miR-335; latter was noted to be responsible for hypoxia-induced proliferation and migration of PASMC [135]. There are no ongoing human clinical trials assessing the role of DCA in treatment of human PAH patients.

**Trapidil** is a vasodilatory agent used in cardiology. Its vasodilatory action is brought about by phosophodiesterase (PDE) and PDGF inhibition. It also has an anti-thrombotic activity by inhibiting TXA_2_ [136]. MCT rat models showed improvement in right ventricular hypertrophy with trapidil administration, with reduction in oxidative stress (reduced NADPH oxidases and an increased ratio of reduced glutathiones/total glutathiones) [137]. Human clinical trials have not been performed on trapidil.

**Nitrite** anion (NO_2_^−^) has been recognized as a source of nitric oxide outside the conventional arginine-NOS pathway, which is generally dysfunctional in PAH. In addition, multiple nitrite reductases have been observed that can convert anion nitrite into nitric oxide, which acts as a pulmonary vasodilator [138]. Simon et al. evaluated the safety and efficacy of inhaled nitrite in 10 patients with PH due to heart failure with preserved ejection fraction, 20 patients with group 1 PH (PAH) and 6 patients with group 3 PH. While inhaled nitrite improved pulmonary hemodynamics in patients with group 2 and group 3 PH, no significant change in pulmonary vascular resistance was noted for group 1 PH patients. Authors concluded that efficacy of nitrite might therefore be limited in PAH patients (NCT01341313) [139].

**Coenzyme Q** (**CoQ**) is an electron carrier in the inner mitochondrial membrane and is essential for proper mitochondrial oxidative metabolism. A single-center clinical trial at the Cleveland Clinic, Ohio, USA, evaluated the role of CoQ in the treatment of PAH. The study enrolled 15 patients with PAH (8 in study group and 7 in control group) that were randomized to receive 12 weeks of oral CoQ supplementation. The researchers monitored cardiac function by echocardiogram and mitochondrial function by heme synthesis and cellular metabolism. While ventricular hemodynamics were improved, there was no significant change in the 6MWD and BNP levels (NCT01148836) [140].

**Xanthine oxidase** (**XO**) catalyzes the conversion of hypoxanthine to xanthine and then xanthine to uric acid. In this reaction, it also generates peroxynitrite and superoxide anions, which are mediators of oxidative damage in PAH. Animal models have corroborated this pathobiologic premise. Higher levels of XO activity in the lungs and in plasma have been reported in hypoxic rat models. These animal studies have concomitantly also reported higher levels of phosphotidylcholine, which is a marker of oxidative stress [141]. While assays of XO activity in PAH is not done, elevated levels of uric acid commonly observed in patients with PAH suggest a hyperactive XO pathway [142,143]. In fact, Spiekermann et al. measured XO activity in the plasma of 31 treatment-naïve iPAH patients at baseline and then in three of these patients three months after treatment with bosentan. In the baseline assay, iPAH patients were associated with higher XO activity levels but these higher levels were not well correlated with pulmonary and cardiac hemodynamic parameters. However, on enzymatic activity analyses of iPAH patients after treatment with bosentan, the XO activity levels were markedly lower than what were initially measured [144]. **Allopurinol** is a XO inhibitor, however despite strong preclinical data, clinical trials have not yet been devised to assess the role of allopurinol in PAH.

### 4.7. Extracellular Matrix

Extracellular matrix (ECM) plays a critical role in the pathogenesis of PAH. In fact, ECM remodeling is said to be a causal factor in PAH rather than a consequence of distal small vessel vasculopathy [145]. Endothelial dysfunction and inflammation disturb the balance between the proteolytic enzymes and their inhibitors, resulting in increased pulmonary vascular resistance and decreased pulmonary arterial compliance (PAC). PAH involves ECM expansion in all three layers of the vessel wall—intima, media, and adventitia, resulting in vascular fibrosis and decreased arterial wall compliance [146]. Researchers have noted increased vascular collagen deposition and cross-linkage of collagen, resulting in insoluble fibers [147]. Increased fragmentation of the internal elastic lamina points toward increased elastin breakdown in pulmonary vasculature in PAH. In PAH, pulmonary vessels also show increased accumulation of tenascin and fibronectin in the vessel walls [148].

ECM composition is regulated by a balance between the proteolytic enzymes, matrix metalloproteinases (MMPs), serine elastases, lysyl oxidases (LOXs) and the inhibiting enzymes, tissue inhibitors of metalloproteinase (TIMP). PAH animal models have shown increased concentration of MMP, elastases, and LOXs as compared to healthy controls [149]. While inciting events can be numerous, endothelial cell injury and subsequent dysfunction has been deemed a critical initiating factor. PAEC injury and dysfunction results in the loss of the endothelial barrier with resulting increased permeability of the barrier. Increased proteolysis results in subsequent activation of growth factors (fibroblast growth factor, transforming growth factor β), which in turn elicit deposition of tenascin, fibronectin, elastin, and collagen, all of which cause vascular stiffness and loss of vessel wall pliability [150]. Inflammation, as previously described, stimulate activation of MMPs and elastases, and further potentiate the inflammatory insult and cause fibrosis. Additionally, stiff ECM activated YAP/TAZ (Yes-associated protein/transcriptional coactivator with PDZ-binding motif) through mechanotransduction. The YAP/TAZ pathway further stimulates proliferation of PAEC and PASMC [151].

MMP-2 and 9 are the major enzymes in the pulmonary vasculature. MMP inhibition has shown clinical benefit in MCT-induced rodent models [11]. Intrathecal instillation of the human TIMP-1 gene in MCT rodent models improved pulmonary hemodynamic and right ventricular hypertrophy [152]. In fact, amlodipine was shown to inhibit MMP-2 activity in MCT rat models, and showed improvement platelet activation, PAEC injury, and PASMC proliferation [153,154,155]

**Elafin** is a serine elastase inhibitor. Several initial studies on PAH noted increased elastase activity in PH experimental models [156,157,158,159]. Hypoxic mice models overexpressing elafin were observed to be protected from PH [160]. Elafin is postulated to protect against PH via suppression of MMP -9. Additionally, Nickel et al. reported alleviation of PH in sugen/hypoxia rats via elafin as evidenced by improvements in occlusive pulmonary arteriopathy, right ventricular hypertrophy, and right ventricular end systolic pressures. Researchers reported that elafin increased apelin activity in PAEC, promoted PASMC apoptosis, and decreased neointimal lesions in lung vasculature. Elafin also stabilized endothelial caveolin-1, subsequently enhancing BMPR2-caveolin-1 interaction. The latter was reported to improve PH phenotype via BMPR2 signaling [161]. In a recent observational cohort study recruiting 249 PAH patients and 106 healthy controls, neutrophil elastase and elafin levels were measured in all the participants. Blood levels of elastase were elevated and elafin were diminished across all PAH subtypes. Higher levels of elastase correlated with more severe disease, poorer functional status (assessed via 6MWD), greater right ventricular dysfunction, elevated cytokines, and lower BMPR2 blood levels. Furthermore, higher elastase levels were also associated with poor clinical outcomes [162]. Currently, a multiple-ascending-dose, randomized, placebo-controlled, blinded clinical trial is underway that aims to evaluate the safety, tolerability, and pharmacological parameters of subcutaneous elafin therapy in healthy adult subjects. The study aims to assess elafin that is being developed as a therapeutic option in PAH (NCT03522935).

### 4.8. Metabolic Pathway

An association has been found between glucose intolerance and insulin resistance. A causal relationship between insulin resistance and PAH has been suggested based rodent models with BMPR2 mutations developing insulin resistance early in their disease process [163]. While it is known that glucose intolerance is a predictor of mortality in PAH, it is unknown if glucose intolerance plays a role in PAH pathogenesis. Several key PAH associated conditions (connective tissue diseases, HIV, and stimulant use) also have an association with insulin resistance [164].

**Metformin**, a biguanide oral anti-hyperglycemic agent used to reduce insulin resistance, has been shown to have a beneficial effect on the NO, prostacyclin, and endothelin signaling pathways in PAH. The proposed mechanism for this effect takes place through activation of the AMP-Kinase which inhibits the proliferation of PASMCs induced by ET-1 [165]. Metformin has also been linked to a host of separate pathways in PAH, including blocking the estrogen pathway through inhibiting aromatase transcription, decreasing Rho-kinase activity, and increasing prostacyclin synthase activity [11,164,165,166,167]. A phase 2 trial with metformin in PAH patients in 2020 found metformin to be safe and well-tolerated in these 20 patients, and suggested metformin may be associated with improved RV function [168]. Another phase 2 trial is underway in PAH assessing hemodynamics and 6MWD in 130 participants (NCT03617458).

Peroxisome proliferator-activated receptor-γ (PPAR-γ) pathway dysregulation has been implicated in PAH pathogenesis. PPAR-γ is a nuclear transcription factor involved in lipogenesis and energy metabolism, adipocyte differentiation, inflammation mediation via downregulation of inflammatory cytokines, anti-cancer phenotype, and modulation of neurodevelopmental disorders [169]. Decreased PPAR-γ levels have been observed in PAH patients, with normal levels in healthy controls and patients with other lung diseases such as chronic obstructive pulmonary disease (COPD) [170]. Downregulation of PPAR-γ has been implicated in inappropriate cell cycle regulation, ubiquitin-mediated DNA repair pathways, and endothelial cell proliferation via VEGF dependent mechanisms [10]. PPAR-γ has been described as a master regulator in BMP2 and TGFβ pathways in PASMCs [171]. In fact, in non-ischemic left ventricular failure, deletion of PPAR-γ cofactor and PPAR-γ coactivator 1α resulted in increased glucose oxidation, decreased fatty acid oxidation, and overall worsening of cardiac function [172]. Thiazolidinediones, PPAR-γ agonists, are a group of oral anti-hyperglycemia agents described for their use in the treatment of diabetes mellitus. Two major approved drugs in this class are pioglitazone and rosiglitazone.

**Pioglitazone** has been shown to improve PAH phenotype in experimental animal models. Lagchenko et al. observed dysregulated cardiac hypertrophy, fibrosis, fatty acid oxidation, and TGF-β signaling in RV failure in Sugen/hypoxia rat models. Reversal of these changes was demonstrated with pioglitazone. In fact, mitochondrial disarray and increased intra-myocellular lipids observed in failed right ventricular cardiomyocytes were also noted to diminish with pioglitazone. In addition, pioglitazone was also demonstrated to curb the elevated levels of miR-197 and miR-146b in these animal models, further supporting the role of microRNAs in the pathogenesis of PAH while providing a therapeutic pathway to counteract them [171]. A clinical trial devised to compare the therapeutic effects of pioglitazone vs. established PAH therapy (bosentan) was terminated due to poor trial enrollment (NCT00825266).

**Rosiglitazone** has been evaluated in preclinical experimental settings. In MCT-induced PH rat models, rosiglitazone was shown to significantly reduce right ventricular systolic pressure with partial mitigation of pulmonary vascular remodeling [173]. In a similar study with MCT rat models, Xie et al. showed a reduction in right ventricular hemodynamics with rosiglitazone treatment. Authors also reported increased PTEN expression with decreased Akt phosphorylation associated with rosiglitazone use, suggesting the action of the PPAR-γ agonists on PTEN/PI3K/Akt pathway [174].

With said preclinical evidence of use of thiazolidinediones in PAH, future human clinical trials would be required to evaluate their safety and efficacy in humans.

### 4.9. Neurohormonal Pathways

The neurohormonal system works to maintain cardiac-homeostasis through two main pillars, the autonomic nervous system (ANS) and the renin–angiotensin–aldosterone system (RAAS). The ANS can be subdivided into the parasympathetic nervous system (PNS) and the sympathetic nervous system (SNS) while the RAAS has a classical and alternative pathway. The ANS works to maintain short term control over cardiac homeostasis while the RAAS works over longer periods. PAH is generally associated with an increase in SNS activity, which in turn downregulates PNS activity [175,176]. Locally, β-adrenoreceptors are downregulated in the right ventricle but not the left ventricle in PAH patients [177]. This effect is mediated through atrial and ventricular stretch receptors, key activators of the SNS. Activation of these stretch receptors in the myocardium allows for the increase in contractility needed by the RV to cope with an increased load, but long-term stimulation of these receptors results in downregulation of the β1-adrenoreceptors [177]. Downregulation leads to both atrioventricular uncoupling in the right heart due loss of contractile reserve and RV diastolic dysfunction through decreased protein kinase A activation of titin, a sarcomere protein responsible for the regulation of myocardial stiffness [175,177,178]. Pulmonary vascular remodeling has also been implicated by sustained SNS activation [175]. Impaired β-adrenoreceptor signaling results in vasoconstriction through decreased NO production [179]. Unchecked, this vasoconstriction leads to PASMC hypertrophy and proliferation [180].

Imbalances in the RAAS system are also observed, as levels of angiotensin (Ag) I and II are increased in patients with progressive PAH vs. healthy controls [181]. This increase is not seen in stable PAH, so an association has been made between increased Ag levels and PAH disease progression [181]. Interestingly, the main hormones in the alternative RAAS pathway, Ag1-7 and Ag1-9, are decreased in PAH according to a 2020 study with 85 PAH patients [182]. Ag1-7 has known vasodilatory, antiproliferative, antifibrotic, and antihypertrophic properties, and is believed to be protective of the maladaptive changes in PAH. The angiotensin converting enzyme (ACE)-2 plays a regulatory role in enhancing the alternative RAAS pathway by converting the products of the classical pathway to those of the alternative pathway [182]. However, data remains inconclusive regarding whether the lower levels of ACE-2 reported in PAH intensify the action of RAAS classical pathways over the alternative pathway [179,181,182]. Thus, the classical RAAS and Ag II have been implicated in the pathogenesis of PAH while the alternative RAAS with Ag1-7 and ACE-2 have shown to have protective effects against these changes.

#### 4.9.1. Beta-Blockers

Beta-blockers (β-blockers) directly counteract the effects of the SNS at the β-adrenoreceptors. Though this action seems favorable, the current 2022 ESC/ESR PH guidelines do not recommend their use in PAH [3]. This is based on the idea that the negative ionotropic and chronotropic effects of β-blockers would result in hypotension and decreased exercise capacity in PAH, and this has, in fact, been reported in porto-pulmonary PH [179,183]. While in the advanced stages of PAH, β-blockers may impede the adrenergic system’s ability to counteract low cardiac output.

However, in the early stages of the disease, mediating the SNS could work to prevent RV remodeling [184]. A large propensity study review in 582 iPAH patients showed favorable tolerance to β-blockers [185]. A 2011 study with 6 PAH patients showed that **carvedilol** was safe and well-tolerated in patients also on vasodilators. The study also showed an increase in RV ejection fraction and stroke volume [186]. **Bispropolol** has been shown to reduce RV failure in experimental mouse models [187]. A 2016 study with bispropolol found the drug to be well tolerated and safe in iPAH but showed no benefit at 6 months [188]. Given this evidence regarding the safety of certain β-blockers in PAH, more evidence is warranted to confirm their role in PAH management.

#### 4.9.2. Alternate RAAS Pathway

A promising target under investigation is the alternate hydrolysis of Ag II to Ag 1-7 via ACE-2, as Ag 1-7 has vasodilatory, anti-inflammatory, and anti-fibrotic effects on PAECs [181,182]. Infusion of rhACE2 (GSK2586881)—a recombinant human ACE—in mouse models has shown to improve RV hypertrophy and hemodynamics in PAH [181]. A pilot study with rhACE2 in 5 PAH patients showed safety and tolerability along with improved cardiac output, PVR, and serum creatinine [181,182]. A 2022 study examined the hemodynamic effects in PAH patients after administration of one dose of rhACE2, but showed no significant change in pulmonary vascular resistance, mPAP, or cardiac index. However, the authors opine that a single dose of rhACE might be inadequate to determine the potential benefits of this therapy, particularly in terms of improvement of chronic vascular remodeling [189]. Another clinical trial with rhACE2 in PAH is predicted to be completed in 2023 (NCT01884051).

#### 4.9.3. Mineralocorticoid Receptor Antagonists

Aldosterone is a steroid hormone involved in the RAAS that binds to mineralocorticoid receptors in the heart, kidney, and pulmonary vasculature [190,191]. Elevated levels of aldosterone are seen in PAH due to dysfunction in the RAAS, which leads to elevated levels of Ag II that in turn directly stimulates aldosterone production in the adrenal gland and pulmonary vasculature [190]. Elevated levels of aldosterone activate the mineralocorticoid receptors in cardiac cells and pulmonary vasculature to promote vascular remodeling and consequent RV dysfunction in PAH [190,192]. In the pulmonary vasculature, aldosterone facilitates activation and vascular infiltration of monocytes, macrophages, and lymphocytes, which promote inflammation. Hyperaldosteronism promotes vascular stiffening in PAH by directing insertion of epithelial sodium channels into the endothelial cell membranes [190]. This increases cellular water retention, cellular swelling, and consequent pulmonary arterial stiffness. In the heart, aldosterone increases cardiac NADPH oxidase activity, leading to myocardial endothelial dysfunction. Increased levels of type I and II collagen led to enhanced myocardial stiffness. Aldosterone levels have also correlated positively with pulmonary vascular resistance and inversely with cardiac output in a subset of patients with PAH who had severe disease [193].

Mineralocorticoid receptor antagonists (MRA) have long been used to manage symptoms of RV failure in PAH due to their diuretic properties. The MRAs may also have a separate benefit in the heart and lungs in regulation of the RAAS. Though this class of medications has been observed to be safe and well tolerated in PAH, the efficacy and benefit in human trails with PAH is currently limited. In PH induced mice, the MRA **spironolactone** prevented vascular remodeling in mice induced by MCT and decreased RV systolic pressure and PVR in mice with pre-existing PH [194]. To characterize the efficacy of MRAs in PAH, a retrospective analysis of patients on spironolactone that were enrolled in the ARIES-1 and -2 trials observed that spironolactone use enhanced the benefits of ambrisentan [189,194]. An ongoing clinical trial investigating spironolactone in PAH is currently underway (NCT01712620).

#### 4.9.4. Vasoactive Intestinal Peptide

Vasoactive intestinal peptide (VIP) is a neuroendocrine hormone with potent vasodilatory properties [89]. This hormone also inhibits platelet activation and PASMC proliferation. Hemodynamically, VIP can reduce pulmonary and systemic vascular resistances [195]. VIP binds to hormone specific receptors found on the cell surfaces of airway epithelia, on macrophages surrounding capillaries, and in the subintima of pulmonary arteries and veins [196]. Low levels of VIP have been seen in both the serum and lungs of PAH patients [197]. In a pilot study, 9 patients with PAH were given inhaled VIP (**Aviptadil**) and showed an increase in cardiac output and improved PVR [198]. A later phase II trial with Aviptadil in 56 PAH patients failed to show any clinical benefit [197,198]. A sub-cutaneous formulation was subsequently designed given the concern of negative alteration of results with the inhaled route of medication administration. The efficacy and safety of the inhaled formulation is currently being evaluated in a clinical trial (NCT03315507).

### 4.10. Serotonin Signaling

Serotonin (5-HT) is a vasoactive monoamine that plays numerous roles in the body, including acting as a vasoconstrictor in peripheral circulation. This property has led to 5-HT being implicated in PASMC proliferation through excessive vasoconstriction [28]. The molecule is typically stored in platelets and the endothelium to maintain low serum levels of 5-HT. Low platelet storage and high serum levels of 5-HT are observed in PAH, leading to maladaptive vasoconstriction and PASMC proliferation [11].

Based on this pathophysiologic basis, a 5-HT receptor inhibitor, **terguride**, was studied in a phase II study in PAH patients [37,89]. The study showed no benefit with terguride administration, possibly due to the fact that terguride is selective for the 5-HT_2A_ and 5-HT_2B_ receptors, and the 5-HT_1B_ receptor is the most highly expressed in pulmonary circulation [37]. **Rodatristat ethyl** is a selective inhibitor of tryptophan hydroxylase 1, the rate limiting enzyme in 5-HT biosynthesis. Lazarus et al. began enrollment into a phase IIb study (ELEVATE2) in 2021 evaluating the safety and efficacy of the drug in 90 PAH patients [199,200]. Of note, trials with the selective 5-HT reuptake inhibitors escitalopram and fluoxetine were started in 2008, but the trials were terminated without any publication of the results [28,89]. An analysis from REVEAL data showed an increased mortality and clinical worsening in PAH but was not properly adjusted for cofounders [89].

### 4.11. Estrogen Pathway

Sex hormones have long thought to play a large role in PAH pathogenesis due to epidemiological data. PAH is more commonly diagnosed in women than men at a ratio of almost 4:1 [11]. This ratio is true in both hPAH and iPAH, but as women also tend to have better RV function and increased survival rates compared to men, this phenomenon is described as the “estrogen paradox” [11,89]. Women also seem to respond better to PAH directed therapies [201,202]. The role of estrogen (E2) and its metabolites is of great concern, as some metabolites have proliferative effects on PASMCs and others have anti-proliferative and anti-inflammatory effects. High E2 levels in males is associated with PAH, but has also been seen to have protective effects on the right ventricular and pulmonary artery remodeling seen in PAH through PA vasodilatory effects [166,201]. E2 can also inhibit BMPR2 expression and BMP signaling [202]. Mouse models have also shown that estrogen preserves RV mitochondrial oxidative capacity, which protects the RV from dysfunction in severe PH [202]. A 2016 case-control series made an association between PAH observed in postmenopausal women and in men to have elevated circulating levels of estrogen and lower levels of dehydroepiandrosterone sulfate (DHEA-S) than premenopausal female and male controls [166,203]. Higher serum levels of estrogen were associated with worse six-minute walk distances while elevated levels of DHEA-S were linked with lower right atrial pressure and PVR [11,203].

**Anastrozole** is an FDA-approved nonsteroidal antiestrogen agent used in women in the treatment and prevention of estrogen receptor-positive breast cancer by inhibiting the enzyme aromatase. Preventing the conversion of androgens to E2 with the aromatase inhibitor has been shown to suppress PAH in animal models [204]. A small 2016 study in 18 PAH patients showed anastrozole did significantly reduce E2 levels over 3 months, but did not have meaningful change in hemodynamics. The drug was found to be safe and well tolerated in this “proof of concept” study [205]. These results lead to the current PHANTOM trial, a phase II RCT, double-blind placebo-controlled multicenter trial assessing 6MWD change in 84 PAH patients with anastrozole administered over 12 months (NCT03229499). A small proof of concept trial of 5 PAH patients was recently completed with **Fulvestrant**, a similar estrogen receptor antagonist used in postmenopausal breast cancer, which showed increased 6MWD and stroke volume [11,166]. **Tamoxifen**, another selective estrogen receptor inhibitor, is also currently under investigation in a single center, 24-week RCT examining clinical worsening via 6MWTD and echo parameters (NCT03528902). Although anastrozole seems to be more effective based on limited data, tamoxifen may be safer in pre-menopausal women [201,206].

**Dehydroepiandrosterone**, **DHEA**, is a steroid hormone precursor for both estrogen and testosterone. Lower levels of DHEA have been associated with increased risk of PAH in both men and women. DHEA supplementation significantly improved 6MWD and hemodynamics in a small trial in Group 3 PH in 2012 [207]. There is a current crossover study in PAH patients evaluating the role of DHEA on RV longitudinal strain on cardiac MRI (NCT03648385).

### 4.12. Iron Deficiency

The pathophysiologic role played by iron deficiency in heart failure has been long described. However, iron deficiency is not rigorously documented in right ventricular failure. Approximately 40–60% of patients with PAH have iron deficiency, regardless of anemia, that has correlated with poorer functional and clinical outcomes in these patients [208]. Hypoxic vasoconstriction and elevation of pulmonary artery systolic pressure in PAH induces release of erythropoietin (EPO) that restores oxygen delivery and recompenses the alveolar hypoxia. Infusion of deferoxamine, an iron chelator, in healthy individuals mimicked hypoxia by elevating right ventricular end systolic pressure, and it therefore points toward a role of iron deficiency in pathogenesis of the said hypoxic pulmonary vasoconstriction [209]. Deferoxamine has also been observed to induce the release of EPO [210]. Rat models with an iron-deficient diet have demonstrated deleterious pulmonary vascular remodeling [211]. Several studies have reported an abnormal elevation of hepcidin in iPAH, resulting in the resistance of response to oral iron supplementation. Hepcidin elevation also inhibits intracellular iron export leading to intracellular iron overload, which further promotes mitochondrial dysfunction and oxidative insults. These further cascade into development of lipid peroxidation, DNA oxidation, and protein denaturation, all participating in PAH pathogenesis [212,213]. Zou et al. performed a bioinformatic analysis of iron metabolism related genes (IMRGs) and differential expression of IMRGs (DEIMRGs) in iPAH patients. They observed that the DEIMRGs were upregulated in oxidative stress on Gene Ontology (GO) analysis. Kyoto Encyclopedia of Genes and Genomes (KEGG) analysis ascertained ferroptosis, fluid shear stress, and atherosclerosis pathway to be engaged in regulation of DEIMRGs [214].

Howard et al. evaluated the safety and efficacy of parenteral iron therapy in two randomized, double-blind, placebo-controlled, 12-week crossover studies in PAH patients (NCT01447628). At 12 weeks, researchers noted no significant change in exercise capacity (6MWD), cardiopulmonary hemodynamics, cardiac magnetic resonance imaging or plasma NTpro-BNP [215].

### 4.13. Deoxyribonucleic Acid (DNA) Damage

PAH pathogenesis due to inflammation and consequent DNA damage has been long described. In fact, in the setting of oxidative damage, PAEC and PASMC demonstrate an oncologic phenotype with augmented proliferation, migration, endothelial-to-mesenchymal transition, and fibrosis. Late in the 1990s, it was discovered that the cells in the pulmonary endothelial plexiform lesions were monoclonal, arising from a single endothelial cell with dysregulated DNA mitogenesis [216]. In-vitro studies of human PAH pulmonary arteries and human-PASMC displayed increased concentrations of DNA damage markers (53BP1 and γ-H2AX) and poly (ADP-ribose) polymerase-1 (PARP-1), a DNA repair enzyme [217]. PARP1 expression has been hypothesized to allow persistent proliferation of cells despite incurring DNA damage along with activation of nuclear factor of activated T-cells (NFAT) and HIF-α. The latter two mediators have been extensively described to play critical roles in the PAH disease process, with inappropriate PASMC proliferation and apoptosis resistance [218]. In vivo MCT rat model experiments demonstrated mitigation of the PAH endophenotype with PARP inhibition using ABT-888 [217]. Federici et al. measured DNA damage, mutagen sensitivity, and ROS in lung and blood cells in patients with PAH. Researchers noted increased baseline DNA damage in PAECs and peripheral blood mononuclear cells (PBMCs) of PAH patients (*p* < 0.05 and *p* < 0.001). In addition, relatives of patients with PAH also demonstrated a similar increase, indicating a role of DNA damage might precede PAH development [219]. Vitry et al. reported that Nudix hydrolase (NUDT1) is a key enzyme that protects against DNA damage in pulmonary vasculature and might play role in PAH pathogenies [220].

**Olaparib** is an FDA approved treatment of BRCA mutation positive ovarian cancer. Olaparib is an oral PARP enzyme group inhibitor. Preclinical findings theoretically support the idea of a PARP inhibitor being beneficial in PAH treatment. In a relatively small clinical trial, a Canadian group of researchers have recruited 20 patients with PAH, already on established PAH therapies for at least 4 weeks (NCT03782818). The patients would be started on a gradual dose escalation regimen of olaparib with outcomes measured at 24 weeks. The study has completed recruiting patients and is currently underway.

### 4.14. FoxO1 Pathway

Autophagy is an important, closely regulated, evolutionarily conserved catabolic process that plays a critical role in cell survival, turnover, and internal cellular homeostasis. Multiple reports in the literature describe dysregulation of autophagy and the role it plays in the pathogenesis of PAH. A higher concentration of autophagosomes has been observed in human PAH lung samples [221]. MCT rat models demonstrated development of PAH being associated with increased lung expression of LC3B-II, ATG5, and p62, all of which are autophagy markers. FoxO1 is a transcription factor belonging to the forkhead O family. FoxO1 expression is observed to be diminished in patients with PAH and in MCT experimental models [49]. Additionally, FoxO1 has been described to be an important regulator in activation of autophagy [222,223]. It was therefore theoretically proposed that FoxO1 stimulation in PAH can alleviate some of the disease phenotype.

**Paclitaxel** is an anti-microtubule chemotherapy agent used in the treatment of multiple cancers. Paclitaxel has been shown to stimulate FoxO1 transcription factor. In addition, paclitaxel also activates BMPR2 signaling, which is frequently dysregulated in PAH. Treatment of MCT rats with paclitaxel showed an increased FoxO1 expression and restoration of autophagy. It also reduced both the RV end systolic pressure and the RV hypertrophy index in these animals [221]. Human clinical trials have not evaluated the role of paclitaxel in treatment of PAH. 

## 5. Interventional Modalities

### 5.1. Balloon Pulmonary Angioplasty

Balloon pulmonary angioplasty (BPA) is an interventional procedure chosen in patients with chronic thromboembolic pulmonary hypertension (CTEPH) who are poor surgical candidates for pulmonary endarterectomy (PEA) or have residual PH after a PEA. The very first case where BPA was initially performed for CTEPH was reported by Voorberg et al. in 1998 [224]. Following a case series by Feinstein et al. describing BPA in 18 patients with CTEPH, wherein despite immediate post-procedure improvement in pulmonary hemodynamics, most of the patients experienced a reperfusion pulmonary edema (RPE) following the procedure, BPA fell out of favor [225]. However, with newer case reports in the last decade showing successful use of BPA despite the potential for complications, ESC/ERS guidelines approved BPA as a treatment for CTEPH under a stringent set of conditions [3].

Unlike PEA, BPA does not extract the clot but rather dilatates chronically obstructed vessels with the inflation of a balloon, causing the fibrin to be crushed to one side of the vessel wall. The web lesions are speculated to be opened via dissection of vascular media and compression of the fibrin while band lesions were enlarged via dilatation of pre-existing central lumen. With multiple obstructions in pulmonary vasculature, staged procedures with repeated dilatations are required [226]. Immediate hemodynamic benefits of BPA are widely reported, with improvement in PVR, mPAP, cardiac index, BNP and 6MWD [227,228,229]. There have been reports of increased oxidative and inflammatory stress post BPA, but it was limited to the first 24 h in the post procedural period [230]. Furthermore, right ventricular hemodynamics were also improved with BPA, such as right ventricular end-diastolic and end-systolic volume index, RV ejection fraction, and RV mass [226]. Long-term effects have not yet been robustly evaluated for BPA. Small-scale studies have reported favorable data, but limited sample size limits the external validity of these studies [226]. Data from Okayama Medical Center in Japan, the world’s largest BPA center, report 1-, 3-, 5-, and 10-year survival rates at their center at 98.6%, 94%, 92.5%, and 89.5%, respectively [231].

Initial reports of BPA had a significantly high incidence of RPE, but with the advent of procedural innovations, advanced imaging techniques assisting in BPA, and improved post-procedural care, RPE is now a rare complication of BPA [226]. The most common injury after BPA is lung injury, noted in 46% of cases [232]. An encouraging body of evidence exists for BPA, and in the correct setting with a multidisciplinary team, it can be an attractive option, especially for the elderly, or patients with surgical contraindications [233].

### 5.2. Pulmonary Artery Denervation

The role of the autonomic nervous system in the etiopathogenesis of PAH has been described in multiple reports [234,235,236]. PAH patients are observed to have a higher sympathetic tone, tachycardia, and pronounced activation of the renin-angiotensin-aldosterone axis. These result in a vasoconstrictive response by the arterioles with associated vascular remodeling [237].

The pulmonary artery is richly supplied with sympathetic fibers, with the bulk located at the left lateral wall near to the main pulmonary artery trunk, close to the ostium of the left pulmonary artery [238]. Preclinical studies have reported an improvement in hemodynamics with targeted pulmonary artery denervation (PADN). Chen et al. demonstrated resolution of balloon-occlusion-induced acute pulmonary hypertension changes (elevated mPAP, PVR, and mean right ventricular pressure) with pulmonary artery denervation in Mongolian dogs [239]. A similar improvement in pulmonary hemodynamics with PADN was also observed with thromboxane A_2_ induced PAH in porcine models [240]. In 2013, Chen et al. reported the data from a clinical trial evaluating the safety and efficacy of PADN (PADN-1 STUDY) in the first-ever human clinical trial. The researchers enrolled 21 patients with iPAH, 13 of which underwent PADN at the main pulmonary trunk and right and left pulmonary artery ostia. The patients who underwent PADN had a significant reduction in mPAP with a significant improvement in 6MWD at the 3-month follow-up [241]. Similar results have also been reported for PADN in PAH due to combined pre- and post-capillary pulmonary hypertension [242]. In a pivotal paper by Zhou et al., authors induced PH in dogs by intra-atrial injection of dehydrogenized-monocrotaline. The experimental animals with PH had significantly thicker nerve myelin sheaths with greater mean axon diameters as compared to controls. PADN induced significant sympathetic nerve demyelination, decreased axonic diameter, and subsequently reduced nerve conduction velocity. These experimental models also showed significant improvement in PH hemodynamics (mPAP, PVR, medial wall thickness) [238,243].

PADN-CFDA trail recently published their results in September 2022. Researchers devised a randomized controlled trial to evaluate the therapeutic effects of PADN in the treatment of PAH. A total of 128 patients with PAH (group 1 PH) who were not on any PAH-specific therapy for at least 30 days were enrolled in the trials and randomized to receive PADN with a phosphodiesterase inhibitor (PDEi) vs. sham therapy with PDEi. The 6-month follow-up reported a significant improvement in 6MWD in the PADN group (mean-adjusted group difference 33.8 m, *p* < 0.001) with a significant reduction in PVR (adjusted difference −1.4, CI −2.6 to −0.2). Furthermore, the PADN group also had improved right ventricular hemodynamics (right ventricular functional improvement, reduction in tricuspid regurgitation, and reduction in NT pro-BNP) [244].

### 5.3. Atrial Septostomy

A theoretical basis exists for creating an atrial septal defect permitting right to left shunting for the treatment of severe PAH. The right to left shunting allows for unloading of the right side of the heart, decreased right ventricular preload, and consequently should improve the right heart dysfunction PAH. With similar levels of mPAP, patients with Eisenmenger’s syndrome have longer survival than patients with primary PAH [245].

The earliest case report trialing atrial septostomy (AS) in an end-stage PAH patient was described by Rich and Lam in 1983. While the patient survived the initial procedure without any additional medical support, she passed away the following morning due to severe systemic hypotension and signs of left ventricular volume overload noted at necropsy [246]. In a retrospective case control study evaluating the outcomes after AS alone versus AS along with other PAH treatment, there were demonstrated improvements in hemodynamic parameters with patients undergoing the procedure that are associated with improved survival (cardiac index, right ventricular filing pressure, 6MWD). However, the progressive decline in survival after 3–5 years after the procedure reinforced the said palliative nature of this intervention [247]. Kurzyna et al. performed balloon atrial septostomy in 11 end-stage PAH patients. The authors observed improved cardiac index and systemic oxygen concentration with transient post-procedural elevation in PVR. Six patients were noted to have spontaneous closure of the atrial defect on mean 12-month follow-up [245].

Yan et al. performed the first in-human modified atrial septostomy combining radiofrequency ablation and balloon dilatation in severe PAH patients. Under echocardiographic guidance, operators ablated fossa ovalis point-by-point by radiofrequency ablation, followed by trans-septal perforation and balloon dilatation, and finally, repeat radiofrequency ablation at the fenestration site. The patients were noted to have a WHO functional class increase by 1 (*p* < 0.0001) and improvement in exercise capacity (+159.5 m, *p* < 0.001). At median 15.5-month follow-up, all intra-atrial connections were patent with stable size [248].

## 6. Newer Emerging Therapies

### 6.1. Immunotherapy

Antibodies targeting molecules intimately involved in disease pathophysiology using immunotherapy and generation of vaccines is a newer approach in disease therapeutics. Endothelin receptor antagonism is an established therapy in PAH. Researchers from China developed a vaccine against second extracellular loop (ECL2) of the endothelin-1 receptor type-A (ETAR). ECL2 plays a role in the activation of ETAR. The vaccine **ETRQβ**-**002** demonstrated good antigenicity with the development of adequate antibodies in vitro. In vivo, the vaccine showed improvement in PAH-related pulmonary vascular remodeling and RV hypertrophy in MCT-rates and Sugen/hypoxia mice [249].

### 6.2. Micro RNAs (miR)

Micro RNAs (miR) are small, non-coding segments of DNA that bind to the 3′ untranslated region of DNA resulting in gene silencing. miR are responsible for gene expression regulation and protein activity in the human body. A significant body of experimental literature regarding in-vitro and in-vivo animal models reports the involvement of miR in the pathogenesis of PAH. Alteration in expression of many miR, such as upregulation of miR-126 and miR-21, has been described in PAH [250].

While still an experimental treatment modality, it is certainly a rational consideration to further evaluate the role of miR in treatment in human PAH. Table 2 describes miR involved in PAH pathogenesis.

### 6.3. Gene Therapy

Mutations in BMPR2 expression and associated downstream signaling has been implicated widely in idiopathic and heritable forms of PAH. As described earlier in this review, BMPR2 gene dysregulation results in inappropriate PASMC and PAEC proliferation, migration, and apoptosis resistance in PAH. In addition to BMPR2, about 450 other gene mutations have been implicated in hereditary PAH (hPAH) [39]. Thus, theoretically, replacing and correcting the mutated gene for treating PAH using viral vectors is a therapeutic possibility. Reynolds et al. transfected in vitro cellular cultures with adenoviral vectors containing BMPR2 gene. These cells showed upregulation of SMAD signaling and decreased cell proliferation. A similar gene delivery to the pulmonary endothelium of rats using adenoviral vectors demonstrated improvements to right ventricular hemodynamics [251].

However, adenoviral vectors induce a strong inflammatory reaction with its gene transfer, thus limiting their feasibility [11]. Gene therapy is a relatively new therapeutic prospect approved for only a handful of disease conditions. Further studies would be necessary to assess the safety, efficacy, and feasibility of this therapy.

## 7. Conclusions

PAH is a chronic progressive cardiopulmonary disease which is uniformly lethal, with development of occlusive proliferative vascular remodeling and associated hemodynamic decompensation. The currently available treatment modalities target only a small aspect of a vast multifaceted disease process which results in the pathophenotype seen in PAH. These therapies also fail to target the underlying pathogenesis of the disease process. Additionally, newer therapeutic options are also becoming available for the management of group 2 PH and CTEPH.

A large body of evidence has described the multiple pathogenetic pathways responsible for PAH genesis, but it is perhaps an arbitrary combination of these pathways in concert with distinct individual genetic phenotypes that cause the final ailment. The preclinical and clinical work done in advancing our knowledge in the past three decades have presented multiple target avenues to combat PAH.

While data exists in support of many of the pathophysiologic pathways underlying the purported novel therapies, most these treatment avenues currently lack robust solid human clinical trial evidence substantiating their use in clinical medicine. We hope with the coming decade, the therapies for PAH continue to evolve with the advent of robust randomized human trials.

## Figures and Tables

**Table 1:** Preclinical treatments targeting oxidative pathway in PAH. Modified and adapted from Xu et al., 2022 [129]. ASK1 = Apoptosis Signal-Regulating Kinase 1, Bax = Bcl-2-Associated X, Bcl2 = B-cell Lymphoma 2, Bsg = Basigin, CAT = Catalase, CGMP = Cyclic Guanosine Monophosphate, Cyp-A = Cyclophilin A, DCA/ATO = Dichloroacetate/Atorvastatin, ENOS = Endothelial Nitric Oxide Synthase, ETA/ETB = Endothelin Receptor A/B, Gp = Glycoprotein, GSH-PX = Glutathione Peroxidase, GSSG = Oxidized Glutathione, HIF1 = Hypoxia-Inducible Factor-1, HO 1 = Heme Oxygenase, ICAM 1 = Intercellular Cell Adhesion Molecule 1, INOS = Inducible Nitric Oxide Synthase, JNK = c-Jun N-terminal Kinase, MCT = Monocrotaline, MDA = Malondialdehyde, MREN2 = Male Transgenic 27 Rat, NADPH = Nicotinamide Adenine Dinucleotide Phosphate Hydrogen, Nf-kb = Nuclear Factor-κB, NFAT = Nuclear Factor of Activated T Cells, NLRP = Nucleotide-Binding Oligomerization Domain, Leucine Rich Repeat And Pyrin Domain Containing, NO = Nitric Oxide, Nox = Nicotinamide Adenine Dinucleotide Phosphate Oxidase, NQO1 = NAD(P)H-Quinone Oxidoreductase-1, Nrf 2 = Nuclear Factor Erythroid 2-Related Factor 2, NT = Nitrotyrosine., Oxr = Oxidation Resistance, PAEC = Pulmonary Artery Endothelial Cells, PAH = Pulmonary Artery Hypertension, PIM 1 = Moloney Murine Leukemia Provirus Integration Site 1, PKG 1 = Cyclic Guanosine Monophosphate-Dependent Protein Kinase Type 1, PVR = Pulmonary Vascular Resistance, ROS = Reactive Oxygen Species, RV = Right Ventricle, SMC = Smooth Muscle Cell, SOD = Superoxide Dismutase, STAT3 = Signal Transducer And Activator Of Transcription 3, SuHx = Sugen Hypoxia, T-AOC = Total Antioxidant Capacity, TBARS = Thiobarbituric Acid Reactive Substances, TGF = Transforming Growth Factor, VDAC = Voltage-Dependent Anion Channels, VPO1 = Vascular Peroxidase 1.

Drugs	Animals (Model)	Biological Indicators	Administration	Observed Effect
Alginate oligosaccharide	Sprague-Dawley rats (MCT, intraperitoneal)	p47-phox, p67-phox, and gp91-phox, subunits of NADPH oxidase, MDA	intraperitoneal	Down-regulate the expressions of malondialdehyde and NADPH by inhibiting the TGF β1/p-Smad2 signaling pathway to prevent the pulmonary vascular remodeling induced by MCT
Vardenafil	Sprague-Dawley rats (MCT, intraperitoneal)	8-iso-prostaglandin-F2a, 3-nitrotyrosine, eNOS, NO, MDA, SOD, Nox2, Nox4	intragluteal	Suppress proliferation of enhanced apoptosis of pulmonary artery smooth muscle cells, attenuating small pulmonary artery remodeling, and right ventricular hypertrophy
Pentaerythritol Tetranitrate (PETN)	Wistar rats (MCT, intravenous)	HO-1, ICAM-1	intragluteal	PETN therapy improved endothelium-dependent relaxation in pulmonary arteries and reduced oxidative stress
Sulforaphane	Male mice (SU5416 and 10% hypoxia, SuHx)	Nrf2, NQO1, NLRP3	intragluteal	Reduce SuHx-induced pulmonary vascular remodeling, inflammation, and fibrosis
Crocin	Sprague-Dawley rats (Hypoxia)	OXR1, P21, Nrf2	intraperitoneal	Crocin co-treatment significantly improved the hemodynamic, oxidative stress biomarkers and histological data of the PAH rats
Melatonin	Newborn sheep (Chronic hypobaric hypoxia, Putre, 3600 m)	SOD2, CAT, GPx1, VDAC, p47-phox, Xanthine Oxidase, 8-isoprostanes, 4HNE, and NT	intragluteal	Reduced major sources of pro-oxidative ROS at the cellular level, reduced oxidative stress and enhanced antioxidant status at the pulmonary level of neonatal PAH
Resveratrol	Sprague-Dawley rats (MCT, hypoxia)	Nrf2, HIF-1 α	intragluteal	Exert antiproliferation, antioxidant, and anti-inflammation effects
Ellagic acid	Male Sprague-Dawley rats (Porcine pancreatic elastase, intra-tracheal)	SOD, CAT, and glutathione	intragluteal	Reduce oxidative stress and prevent PAH
18β-Glycrrhetinic Acid	Male Sprague-Dawley rats (MCT, intraperitoneal)	Nox2, Nox4	intragluteal	Reduce the changes in oxidative stress biomarkers and inhibit Nox2 and Nox4 expression
Celastramycin	Wild-type mice; SD rats (3 wks. of hypoxic exposure; SU5416, subcutaneous)	ROS, Nrf2, Nox, GSH/GSSG, SOD2	Osmotic pump; intraperitoneal	Increase protein levels of Nrf2 and improve pulmonary hypertension
Celastrol	cROCK1-/- and cROCK2-/- mice (TAC)	CyPA, Bsg, Nox2, Nox4	intraperitoneal	Inhibit CyPA/Bsg-NF-ΚB axis and enhance ROS production
Hybridization of Isosorbide 5 Mononitrate and Bardoxolone Methyl	Male Sprague-Dawley rats (MCT, intracerebral)	NO, Nox4	intratracheal	By inactivating Nox4, excessive proliferation of vascular pericytes was inhibited, macrophage infiltration and oxidative stress were reduced, cardiac hypertrophy and fibrosis were significantly reduced in rats with pulmonary hypertension
Combination of DCA/ATO	Male Sprague-Dawley rats (MCT, intracerebral)	CHOP, Bcl2	intragluteal	The combined treatment of DCA/ATO significantly reduces the right ventricular systolic blood pressure accompanied by a decrease in right heart hypertrophy and reduces vascular remodeling, thereby inhibiting excessive PASMC proliferation
Baicalein	Male Sprague-Dawley rats (MCT, intracerebral)	MDA, SOD, GSH-Px, Bax, Bcl-2	intragluteal	Inhibit oxidative stress and alleviated pulmonary vascular remodeling in MCT-induced PAH
17-β estradiol	Male Sprague-Dawley rats (MCT, intracerebral)	T-AOC, MDA, Nox4	intraperitoneal	Inhibit Nox4-mediated oxidative stress and alleviated MCT-induced right ventricular remodeling of PAH rats
Copaiba Oil	Male Wistar rats (MCT, intraperitoneal)	eNOS	intragluteal	Reduce oxidative stress and apoptosis signaling in RV of rats with PAH
Dimethyl Fumarate	Male C57BL/6 mice (Hypoxic chamber)	HO-1, NOX4	intraperitoneal	Mitigate oxidative stress damage and inflammation in lung
Bucindolol	Male Wistar rats (MCT, intraperitoneal)	eNOS, SOD-1	intraperitoneal	Decrease (21%) PVR and increase RV workload, thereby improving the vascular remodeling of the pulmonary artery
Rosuvastatin	Male Ren2 and Sprague-Dawley rats (Transgenic (mRen2) 27 rats)	3-NT, NO(x), Nox, and endothelial NO synthase expression	intraperitoneal	Improve cardiovascular outcomes/risk by restoring endothelial and SMC function, inhibiting SMC proliferation, reducing oxidative stress and inflammation in the vascular wall
Carvacrol	Male Wistar rats (Hypoxia)	SOD, GSH, MDA, caspase 3	intraperitoneal	Attenuate the pulmonary vascular remodeling and promotes PASMC apoptosis
Trapidil	Male Wistar rats (MCT, intraperitoneal)	NADPH oxidases, glutathiones/total glutathiones	intraperitoneal	Improve hemodynamic, echocardiographic, and redox state parameters of right ventricle
Tetrandrine	Male Sprague-Dawley rats (MCT, intraperitoneal)	cGMP, PKG-1, iNOS	intraperitoneal	Alleviate MCT-induced PAH through regulation of NO signaling pathway and antioxidant and antiproliferation effects
Trimethoxystilbene	Male Sprague-Dawley rats (hypoxic chamber)	Nox2, Nox4, VPO1	intragluteal	Attenuate hypoxia-induced pulmonary vascular remodeling and right ventricle hypertrophy accompanied by downregulation of Nox2, Nox4 and VOP1 expression
Hydrogen	Male Sprague-Dawley rats (MCT, intracerebral)	STAT3, NFAT	Housed ad libitumto hydrogen- saturated water	Ameliorate MCT-induced PAH in rats by suppressing macrophage accumulation, reducing oxidative stress, and modulating the STAT3/NFAT axis
Blueberry extract	Male Wistar rats (MCT, intragluteal)	NADPH, SOD, GPx, ETA/ETB	intragluteal	Decrease the mean pulmonary artery pressure and total reactive species concentration and lipid oxidation
Ocimum Sanctum (Linn)	Male Wistar rats (MCT, intracerebral)	Thiobarbituric Acid Reactive Substances (TBARS); GSH; Catalase; SOD; Nox1	intragluteal	Decrease Nox-1 expression and increase expression of Bcl2/Bax ratio caused by MCT
Honokiol	Male Sprague-Dawley rats (MCT, intraperitoneal)	CyPA	intragluteal	Alleviate autophagy and PAH regulated by CyPA in PAECs
GS-444217/Selonsertib	SD rats (MCT (intracerebral)/Sugen/hypoxia)	phosphorylation of p38 and JNK	intragluteal	Reduce pulmonary arterial pressure and RV hypertrophy in PAH models associated with reduced ASK1 phosphorylation, reduced muscularization of the pulmonary arteries, and reduced fibrotic gene expression in the RV
SGI-1776, TP-3654	Male SD rats (MCT, intracerebral/Fawn-Hooded Rats)	Repair of DNA damage	intragluteal	Improve significantly pulmonary hemodynamics (right heart catheterization) and vascular remodeling (Elastica van Gieson)

**Table 2:** Micro RNAs involved in pulmonary artery hypertension pathogenesis. miR = Micro RNA, BMPR-II = Bone Morphogenetic Protein Receptor Type II, MCT = Monocrotaline, NOS = Nitric Oxide Synthase, PAEC = Pulmonary Artery Endothelial Cell, PASMC = Pulmonary Artery Smooth Muscle Cell, PAH = Pulmonary Arterial Hypertension, PH = Pulmonary Hypertension, NFAT = Nuclear Factor Activated T-Cells, VEGF = Vascular Endothelial Growth Factor, RV = Right Ventricle, PPARγ = Peroxisome-Proliferator Activated Receptor Gamma, MCU = Mitochondrial Calcium Uniporter, APLN = Apelin, FGF = Fibroblast Growth Factor, NA = Not Applicable. Arrows indicate increased vs. decreased expression of the microRNA in discussion and thus communicates to its role in the pathogenesis of PAH.

MicroRNA Expression in PAH; Human Model; Animal Model	Effect
miR-17-92 ↑; NA; Mouse-Hypoxia, Rat-monocrotaline, hypoxia	Increased PASMC proliferation, induced by IL-6; overexpression downregulates BMPR-II
miR-21 ↑; Pulmonary arteries, plexiform lesions; Mouse-hypoxia, Sugen5416/hypoxia, *VHL* null Interleukin-6 transgenic, Rat-monocrotaline	Decreased NOS expression in hypoxic PAECs, increased PASMC proliferation; miR-21 deletion enhances PH in mice
miR-126 ↓; Right ventricle; Rat-monocrotaline	Inhibition of VEGF pathway and decrease in RV vascular density
miR-145 ↑; Lung tissue, plexiform lesions; Mouse-hypoxia, BMPR2 mutation	Decrease in miR-145 is protective against hypoxia-induced PAH
miR-150 ↓; Plasma; NA	Associated with poor survival
miR-204 ↓; Lung, pulmonary arteries; Rat-monocrotaline, Sugen5416/hypoxia, Mouse-hypoxia	Increased NFAT, PASMC proliferation; miR-204 mimics prevent PH in monocrotaline model
miR-210 ↑; Pulmonary artery; Mouse-Sugen5416/hypoxia	Inhibits PASMC apoptosis by suppressing E2F3 transcription factor expression
miR-214 ↑; NA; Mouse-hypoxia, Sugen5416/hypoxia, Rat-monocrotaline, Sugen5416/hypoxia	Increased right ventricular hypertrophy in hypoxia models
miR-130/301 ↑; NA; Mouse-hypoxia, Sugen5416/hypoxia, *VHL* null, Interleukin-6 transgenic, BMPR2X transgenic, *Schistosoma mansoni*-infected, Rat-monocrotaline, Juvenile lamb-pulmonary artery-aorta shunt	Increased PAEC proliferation and PASMC contraction via PPAR-γ mediated pathways
miRNA-21 and miRNA-27a ↓; PAECs and PASMCs; NA	Suppress PAEC and PASMC proliferation
miR-26a ↓; PAH patient plasma; Rats-monocrotaline	Inhibition of miR-26a promotes apoptosis of rat cardiomyocytes and pathological right ventricular hypertrophy in PAH
miR-124 ↓; Pulmonary artery smooth muscle cells; Mouse-chronic hypoxia	Suppression of NFAT pathway, antiproliferative
miR-138 and miR-25 ↑; Pulmonary artery smooth muscle cells; Rats-monocrotaline	Downregulation of MCU, increased PASMC proliferation, apoptosis resistance; inhibition of miRs prevent PH in monocrotaline model
miR-140-5p ↓; NA; Rat-monocrotaline, Sugen-hypoxia	Inhibition of miR 140-5p promotes smooth muscle cell proliferation
miR-424/503 ↓; Pulmonary artery endothelial cells; APLN knock-out mice	Reduced endothelial proliferation, decreased expression of FGF-2 and FGF receptor-1; restoration of miRs prevents monocrotaline and Sugen-hypoxia induced PH.

## Data Availability

Not applicable.

## References

[1] Valerio C.J., Schreiber B.E., Handler C.E., Denton C.P., Coghlan J.G. (2013). Borderline Mean Pulmonary Artery Pressure in Patients With Systemic Sclerosis: Transpulmonary Gradient Predicts Risk of Developing Pulmonary Hypertension. Arthritis Rheum..

[2] Goldberg A.B., Mazur W., Kalra D.K. (2017). Pulmonary hypertension: Diagnosis, imaging techniques, and novel therapies. Cardiovasc. Diagn. Ther..

[3] Humbert M., Kovacs G., Hoeper M.M., Badagliacca R., Berger R.M.F., Brida M., Carlsen J., Coats A.J.S., Escribano-Subias P., Ferrari P. (2022). 2022 ESC/ERS Guidelines for the diagnosis and treatment of pulmonary hypertension. Eur. Heart J..

[4] Hoeper M.M., Mayer E., Simonneau G., Rubin L.J. (2006). Chronic Thromboembolic Pulmonary Hypertension. Circulation.

[5] Simonneau G., Montani D., Celermajer D., Denton C.P., Gatzoulis M.A., Krowka M., Williams P.G., Souza R. (2019). Haemodynamic definitions and updated clinical classification of pulmonary hypertension. Eur. Respir. J..

[6] Kovacs G., Berghold A., Scheidl S., Olschewski H. (2009). Pulmonary arterial pressure during rest and exercise in healthy subjects: A systematic review. Eur. Respir. J..

[7] Kovacs G., Douschan P., Maron B.A., Condliffe R., Olschewski H. (2019). Mildly increased pulmonary arterial pressure: A new disease entity or just a marker of poor prognosis?. Eur. J. Heart Fail..

[8] Bae S., Saggar R., Bolster M.B., Chung L., Csuka M.E., Derk C., Domsic R., Fischer A., Frech T., Goldberg A. (2012). Baseline characteristics and follow-up in patients with normal haemodynamics versus borderline mean pulmonary arterial pressure in systemic sclerosis: Results from the PHAROS registry. Ann. Rheum. Dis..

[9] Maron B.A., Hess E., Maddox T.M., Opotowsky A.R., Tedford R.J., Lahm T., Joynt K.E., Kass D.J., Stephens T., Stanislawski M. (2016). Association of Borderline Pulmonary Hypertension With Mortality and Hospitalization in a Large Patient Cohort: Insights From the Veterans Affairs Clinical Assessment, Reporting, and Tracking Program. Circulation.

[10] Shah A.J., Vorla M., Kalra D.K. (2022). Molecular Pathways in Pulmonary Arterial Hypertension. Int. J. Mol. Sci..

[11] Zolty R. (2021). Novel Experimental Therapies for Treatment of Pulmonary Arterial Hypertension. J. Exp. Pharmacol..

[12] Hassoun P.M. (2021). Pulmonary Arterial Hypertension. N. Engl. J. Med..

[13] Vetter N. (2010). Editor’s Choice. Br. Med. Bull..

[14] Huber L., Bye H., Brock M. (2015). The pathogenesis of pulmonary hypertension—An update. Swiss Med. Wkly..

[15] Farber H.W., Loscalzo J. (2004). Pulmonary Arterial Hypertension. N. Engl. J. Med..

[16] Rabinovitch M., Guignabert C., Humbert M., Nicolls M.R. (2014). Inflammation and Immunity in the Pathogenesis of Pulmonary Arterial Hypertension. Circ. Res..

[17] Thenappan T., Ormiston M.L., Ryan J.J., Archer S.L. (2018). Pulmonary arterial hypertension: Pathogenesis and clinical management. BMJ.

[18] Maron B.A. (2020). Pulmonary arterial hypertension: Cellular and molecular changes in the lung. Glob. Cardiol. Sci. Pract..

[19] Wilkins M.R. (2012). Pulmonary hypertension: The science behind the disease spectrum. Eur. Respir. Rev..

[20] Bonnet S., Rochefort G., Sutendra G., Archer S.L., Haromy A., Webster L., Hashimoto K., Bonnet S.N., Michelakis E.D. (2007). The nuclear factor of activated T cells in pulmonary arterial hypertension can be therapeutically targeted. Proc. Natl. Acad. Sci. USA.

[21] Connolly M.J., Aaronson P.I. (2011). Key role of the RhoA/Rho kinase system in pulmonary hypertension. Pulm. Pharmacol. Ther..

[22] Do E.Z., Fukumoto Y., Takaki A., Tawara S., Ohashi J., Nakano M., Tada T., Saji K., Sugimura K., Fujita H. (2009). Evidence for Rho-Kinase Activation in Patients with Pulmonary Arterial Hypertension. Circ. J..

[23] Mair K.M., Yang X.D., Long L., White K., Wallace E., Ewart M.-A., Docherty C.K., Morrell N.W., MacLean M.R. (2015). Sex Affects Bone Morphogenetic Protein Type II Receptor Signaling in Pulmonary Artery Smooth Muscle Cells. Am. J. Respir. Crit. Care Med..

[24] Voelkel N.F., Quaife R.A., Leinwand L.A., Barst R.J., McGoon M.D., Meldrum D.R., Dupuis J., Long C.S., Rubin L.J., Smart F.W. (2006). Right Ventricular Function and Failure. Circulation.

[25] Barst R.J., McGoon M., Torbicki A., Sitbon O., Krowka M.J., Olschewski H., Gaine S. (2004). Diagnosis and differential assessment of pulmonary arterial hypertension. J. Am. Coll. Cardiol..

[26] Sitbon O., Humbert M., Jais X., Ioos V., Hamid A.M., Provencher S., Garcia G., Parent F., Hervé P., Simonneau G. (2005). Long-Term Response to Calcium Channel Blockers in Idiopathic Pulmonary Arterial Hypertension. Circulation.

[27] Rich S., Kaufmann E., Levy P.S. (1992). The Effect of High Doses of Calcium-Channel Blockers on Survival in Primary Pulmonary Hypertension. N. Engl. J. Med..

[28] Zolty R. (2020). Pulmonary arterial hypertension specific therapy: The old and the new. Pharmacol. Ther..

[29] Lavelle A., Sugrue R., Lawler G., Mulligan N., Kelleher B., Murphy D.M., Gaine S. (2009). Sitaxentan-induced hepatic failure in two patients with pulmonary arterial hypertension. Eur. Respir. J..

[30] Lee W.-T.N., Kirkham N., Johnson M.K., Lordan J.L., Fisher A.J., Peacock A.J. (2011). Sitaxentan-related acute liver failure in a patient with pulmonary arterial hypertension. Eur. Respir. J..

[31] Bin-Nun A., Schreiber M.D. (2008). Role of iNO in the modulation of pulmonary vascular resistance. J. Perinatol..

[32] Triposkiadis F., Xanthopoulos A., Skoularigis J., Starling R.C. (2022). Therapeutic augmentation of NO-sGC-cGMP signalling: Lessons learned from pulmonary arterial hypertension and heart failure. Heart Fail. Rev..

[33] Stasch J.-P., Pacher P., Evgenov O.V. (2011). Soluble Guanylate Cyclase as an Emerging Therapeutic Target in Cardiopulmonary Disease. Circulation.

[34] Grimminger F., Weimann G., Frey R., Voswinckel R., Thamm M., Bolkow D., Weissmann N., Muck W., Unger S., Wensing G. (2009). First acute haemodynamic study of soluble guanylate cyclase stimulator riociguat in pulmonary hypertension. Eur. Respir. J..

[35] Ruopp N.F., Cockrill B.A. (2022). Diagnosis and Treatment of Pulmonary Arterial Hypertension. JAMA.

[36] Barst R. (2010). How has epoprostenol changed the outcome for patients with pulmonary arterial hypertension?. Int. J. Clin. Pract..

[37] Sommer N., Ghofrani H.A., Pak O., Bonnet S., Provencher S., Sitbon O., Rosenkranz S., Hoeper M.M., Kiely D.G. (2021). Current and future treatments of pulmonary arterial hypertension. Br. J. Pharmacol..

[38] Maron B.A. (2020). Clarifying the Pulmonary Arterial Hypertension Molecular Landscape Using Functional Genetics. Am. J. Respir. Crit. Care Med..

[39] Fessel J.P., Loyd J.E., Austin E.D. (2011). The Genetics of Pulmonary Arterial Hypertension in the *Post-BMPR2* Era. Pulm. Circ..

[40] Evans J.D.W., Girerd B., Montani D., Wang X.-J., Galiè N., Austin E.D., Elliott G., Asano K., Grünig E., Yan Y. (2016). BMPR2 mutations and survival in pulmonary arterial hypertension: An individual participant data meta-analysis. Lancet Respir. Med..

[41] Happé C., Kurakula K., Sun X.-Q., Bos D.D.S.G., Rol N., Guignabert C., Tu L., Schalij I., Wiesmeijer K.C., Tura-Ceide O. (2020). The BMP Receptor 2 in Pulmonary Arterial Hypertension: When and Where the Animal Model Matches the Patient. Cells.

[42] Morrell N.W., Aldred M.A., Chung W.K., Elliott C.G., Nichols W.C., Soubrier F., Trembath R.C., Loyd J.E. (2019). Genetics and genomics of pulmonary arterial hypertension. Eur. Respir. J..

[43] Nasim T., Ogo T., Chowdhury H.M., Zhao L., Chen C.-N., Rhodes C., Trembath R. (2012). BMPR-II deficiency elicits pro-proliferative and anti-apoptotic responses through the activation of TGFβ-TAK1-MAPK pathways in PAH. Hum. Mol. Genet..

[44] Dewachter L., Adnot S., Guignabert C., Tu L., Marcos E., Fadel E., Humbert M., Dartevelle P., Simonneau G., Naeije R. (2009). Bone morphogenetic protein signalling in heritable versus idiopathic pulmonary hypertension. Eur. Respir. J..

[45] Ali M.K., Ichimura K., Spiekerkoetter E. (2021). Promising therapeutic approaches in pulmonary arterial hypertension. Curr. Opin. Pharmacol..

[46] Spiekerkoetter E., Sung Y.K., Sudheendra D., Scott V., Del Rosario P., Bill M., Haddad F., Long-Boyle J., Hedlin H., Zamanian R.T. (2017). Randomised placebo-controlled safety and tolerability trial of FK506 (tacrolimus) for pulmonary arterial hypertension. Eur. Respir. J..

[47] Chen Y.-F., Feng J.-A., Li P., Xing D., Zhang Y., Serra R., Ambalavanan N., Majid-Hassan E., Oparil S. (2006). Dominant negative mutation of the TGF-β receptor blocks hypoxia-induced pulmonary vascular remodeling. J. Appl. Physiol..

[48] Tielemans B., Delcroix M., Belge C., Quarck R. (2019). TGFβ and BMPRII signalling pathways in the pathogenesis of pulmonary arterial hypertension. Drug Discov. Today.

[49] Long L., Yang X., Southwood M., Lu J., Marciniak S., Dunmore B.J., Morrell N. (2013). Chloroquine Prevents Progression of Experimental Pulmonary Hypertension via Inhibition of Autophagy and Lysosomal Bone Morphogenetic Protein Type II Receptor Degradation. Circ. Res..

[50] Gomez-Arroyo J.G., Farkas L., Alhussaini A.A., Farkas D., Kraskauskas D., Voelkel N.F., Bogaard H.J. (2012). The monocrotaline model of pulmonary hypertension in perspective. Am. J. Physiol. Cell. Mol. Physiol..

[51] Drake K.M., Dunmore B.J., McNelly L.N., Morrell N.W., Aldred M.A. (2013). Correction of Nonsense *BMPR2* and *SMAD9* Mutations by Ataluren in Pulmonary Arterial Hypertension. Am. J. Respir. Cell Mol. Biol..

[52] Siddiqui N., Sonenberg N. (2016). Proposing a mechanism of action for ataluren. Proc. Natl. Acad. Sci. USA.

[53] Mouthon L., Guillevin L., Humbert M. (2005). Pulmonary arterial hypertension: An autoimmune disease?. Eur. Respir. J..

[54] Mukerjee D., George D.S., Coleiro B., Knight C., Denton C.P., Davar J., Black C.M., Coghlan J.G. (2003). Prevalence and outcome in systemic sclerosis associated pulmonary arterial hypertension: Application of a registry approach. Ann. Rheum. Dis..

[55] Nicolls M.R., Taraseviciene-Stewart L., Rai P.R., Voelkel N.F., Badesch D.B. (2005). Autoimmunity and pulmonary hypertension: A perspective. Eur. Respir. J..

[56] Stacher E., Graham B.B., Hunt J.M., Gandjeva A., Groshong S.D., McLaughlin V.V., Jessup M., Grizzle W.E., Aldred M.A., Cool C.D. (2012). Modern Age Pathology of Pulmonary Arterial Hypertension. Am. J. Respir. Crit. Care Med..

[57] Molossi S., Clausell N., Rabinovitch M. (1995). Reciprocal induction of tumor necrosis factor-? and interleukin-? activity mediates fibronectin synthesis in coronary artery smooth muscle cells. J. Cell. Physiol..

[58] Jones P.L., Cowan K.N., Rabinovitch M. (1997). Tenascin-C, proliferation and subendothelial fibronectin in progressive pulmonary vascular disease. Am. J. Pathol..

[59] Terrier B., Tamby M.C., Camoin L., Guilpain P., Broussard C., Bussone G., Yaïci A., Hotellier F., Simonneau G., Guillevin L. (2008). Identification of Target Antigens of Antifibroblast Antibodies in Pulmonary Arterial Hypertension. Am. J. Respir. Crit. Care Med..

[60] Davies R.J., Holmes A.M., Deighton J., Long L., Yang X., Barker L., Walker C., Budd D.C., Upton P.D., Morrell N.W. (2012). BMP type II receptor deficiency confers resistance to growth inhibition by TGF-β in pulmonary artery smooth muscle cells: Role of proinflammatory cytokines. Am. J. Physiol. Cell. Mol. Physiol..

[61] Tian W., Jiang S.Y., Jiang X., Tamosiuniene R., Kim D., Guan T., Arsalane S., Pasupneti S., Voelkel N.F., Tang Q. (2021). The Role of Regulatory T Cells in Pulmonary Arterial Hypertension. Front. Immunol..

[62] Golembeski S.M., West J., Tada Y., Fagan K.A. (2005). Interleukin-6 Causes Mild Pulmonary Hypertension and Augments Hypoxia-Induced Pulmonary Hypertension in Mice. Chest.

[63] Savale L., Tu L., Rideau D., Izziki M., Maitre B., Adnot S., Eddahibi S. (2009). Impact of interleukin-6 on hypoxia-induced pulmonary hypertension and lung inflammation in mice. Respir. Res..

[64] Arita Y., Sakata Y., Sudo T., Maeda T., Matsuoka K., Tamai K., Higuchi K., Shioyama W., Nakaoka Y., Kanakura Y. (2010). The efficacy of tocilizumab in a patient with pulmonary arterial hypertension associated with Castleman’s disease. Heart Vessel..

[65] Taniguchi K., Shimazaki C., Fujimoto Y., Shimura K., Uchiyama H., Matsumoto Y., Kuroda J., Horiike S., Taniwaki M. (2009). Tocilizumab is effective for pulmonary hypertension associated with multicentric Castleman’s disease. Int. J. Hematol..

[66] Kadavath S., Zapantis E., Zolty R., Efthimiou P. (2014). A novel therapeutic approach in pulmonary arterial hypertension as a complication of adult-onset Still’s disease: Targeting IL-6. Int. J. Rheum. Dis..

[67] Toshner M., Church C., Harbaum L., Rhodes C., Moreschi S.S.V., Liley J., Jones R., Arora A., Batai K., Desai A.A. (2022). Mendelian randomisation and experimental medicine approaches to interleukin-6 as a drug target in pulmonary arterial hypertension. Eur. Respir. J..

[68] Lawrie A., Waterman E., Southwood M., Evans D., Suntharalingam J., Francis S., Crossman D., Croucher P., Morrell N., Newman C. (2008). Evidence of a Role for Osteoprotegerin in the Pathogenesis of Pulmonary Arterial Hypertension. Am. J. Pathol..

[69] Voelkel N.F., Tuder R.M., Bridges J., Arend W.P. (1994). Interleukin-1 receptor antagonist treatment reduces pulmonary hypertension generated in rats by monocrotaline. Am. J. Respir. Cell Mol. Biol..

[70] Voelkel N.F., Tuder R. (1994). Interleukin-1 Receptor Antagonist Inhibits Pulmonary Hypertension Induced by Inflammationa. Ann. N. Y. Acad. Sci..

[71] Campos M., Schiopu E. (2012). Pulmonary Arterial Hypertension in Adult-Onset Still’s Disease: Rapid Response to Anakinra. Case Rep. Rheumatol..

[72] Trankle C.R., Canada J.M., Kadariya D., Markley R., De Chazal H.M., Pinson J., Fox A., Van Tassell B.W., Abbate A., Grinnan D. (2019). IL-1 Blockade Reduces Inflammation in Pulmonary Arterial Hypertension and Right Ventricular Failure: A Single-Arm, Open-Label, Phase IB/II Pilot Study. Am. J. Respir. Crit. Care Med..

[73] Fujita M., Shannon J.M., Irvin C.G., Fagan K.A., Cool C., Augustin A., Mason R.J., Eurlings I.M.J., Dentener M.A., Mercken E.M. (2001). Overexpression of tumor necrosis factor-α produces an increase in lung volumes and pulmonary hypertension. Am. J. Physiol. Cell. Mol. Physiol..

[74] Soon E., Holmes A.M., Treacy C.M., Doughty N.J., Southgate L., Machado R.D., Trembath R.C., Jennings S., Barker L., Nicklin P. (2010). Elevated Levels of Inflammatory Cytokines Predict Survival in Idiopathic and Familial Pulmonary Arterial Hypertension. Circulation.

[75] Itoh A., Nishihira J., Makita H., Miyamoto K., Yamaguchi E., Nishimura M. (2003). Effects of IL-1beta, TNF-alpha, and macrophage migration inhibitory factor on prostacyclin synthesis in rat pulmonary artery smooth muscle cells. Respirology.

[76] Chang S.W. (1994). TNF potentiates PAF-induced pulmonary vasoconstriction in the rat: Role of neutrophils and thromboxane A2. J. Appl. Physiol..

[77] Wang Q., Zuo X.-R., Wang Y.-Y., Xie W.-P., Wang H., Zhang M. (2013). Monocrotaline-induced pulmonary arterial hypertension is attenuated by TNF-α antagonists via the suppression of TNF-α expression and NF-κB pathway in rats. Vasc. Pharmacol..

[78] Hurst L.A., Dunmore B.J., Long L., Crosby A., Al-Lamki R., Deighton J., Southwood M., Yang X., Nikolic M.Z., Herrera B. (2017). TNFα drives pulmonary arterial hypertension by suppressing the BMP type-II receptor and altering NOTCH signalling. Nat. Commun..

[79] Mutschler D., Wikström G., Lind L., Larsson A., Lagrange A., Eriksson M. (2006). Etanercept Reduces Late Endotoxin-Induced Pulmonary Hypertension in the Pig. J. Interf. Cytokine Res..

[80] Zhang L.-L., Lu J., Li M.-T., Wang Q., Zeng X.-F. (2019). Preventive and remedial application of etanercept attenuate monocrotaline-induced pulmonary arterial hypertension. Int. J. Rheum. Dis..

[81] Zhang Q., Lenardo M.J., Baltimore D. (2017). 30 Years of NF-κB: A Blossoming of Relevance to Human Pathobiology. Cell.

[82] Sawada H., Mitani Y., Maruyama J., Jiang B.H., Ikeyama Y., Dida F.A., Yamamoto H., Imanaka-Yoshida K., Shimpo H., Mizoguchi A. (2007). A Nuclear Factor-κB Inhibitor Pyrrolidine Dithiocarbamate Ameliorates Pulmonary Hypertension in Rats. Chest.

[83] Price L.C., Caramori G., Perros F., Meng C., Gambaryan N., Dorfmuller P., Montani D., Casolari P., Zhu J., Dimopoulos K. (2013). Nuclear Factor κ-B Is Activated in the Pulmonary Vessels of Patients with End-Stage Idiopathic Pulmonary Arterial Hypertension. PLoS ONE.

[84] Ahmad R., Raina D., Meyer C., Kharbanda S., Kufe D. (2006). Triterpenoid CDDO-Me Blocks the NF-κB Pathway by Direct Inhibition of IKKβ on Cys-179. J. Biol. Chem..

[85] Hsu C.H., Huang W.C., Chang W.T. (2022). Future Perspectives of Pulmonary Hypertension Treatment. Acta Cardiol. Sin..

[86] Grzegorzewska A.P., Seta F., Han R., Czajka C.A., Makino K., Stawski L., Isenberg J.S., Browning J.L., Trojanowska M. (2017). Dimethyl Fumarate ameliorates pulmonary arterial hypertension and lung fibrosis by targeting multiple pathways. Sci. Rep..

[87] Kong K., Koontz D., Morse C., Roth E., Domsic R.T., Simon M.A., Stratton E., Buchholz C., Tobin-Finch K., Simms R. (2021). A pilot study of dimethyl fumarate in pulmonary arterial hypertension associated with systemic sclerosis. J. Scleroderma Relat. Disord..

[88] Zamanian R.T., Badesch D., Chung L., Domsic R.T., Medsger T., Pinckney A., Keyes-Elstein L., D’Aveta C., Spychala M., White R.J. (2021). Safety and Efficacy of B-Cell Depletion with Rituximab for the Treatment of Systemic Sclerosis–associated Pulmonary Arterial Hypertension: A Multicenter, Double-Blind, Randomized, Placebo-controlled Trial. Am. J. Respir. Crit. Care Med..

[89] Bisserier M., Pradhan N., Hadri L. (2020). Current and emerging therapeutic approaches to pulmonary hypertension. Rev. Cardiovasc. Med..

[90] Frantz R.P., Benza R.L., Channick R.N., Chin K., Howard L.S., McLaughlin V.V., Sitbon O., Zamanian R.T., Hemnes A.R., Cravets M. (2021). TORREY, a Phase 2 study to evaluate the efficacy and safety of inhaled seralutinib for the treatment of pulmonary arterial hypertension. Pulm. Circ..

[91] Klein M., Schermuly R.T., Ellinghaus P., Milting H., Riedl B., Nikolova S., Pullamsetti S.S., Weissmann N., Dony E., Savai R. (2008). Combined Tyrosine and Serine/Threonine Kinase Inhibition by Sorafenib Prevents Progression of Experimental Pulmonary Hypertension and Myocardial Remodeling. Circulation.

[92] Kimura G., Kataoka M., Inami T., Fukuda K., Yoshino H., Satoh T. (2017). Sorafenib as a potential strategy for refractory pulmonary arterial hypertension. Pulm. Pharmacol. Ther..

[93] Dhoble S., Patravale V., Weaver E., Lamprou D.A., Patravale T. (2022). Comprehensive review on novel targets and emerging therapeutic modalities for pulmonary arterial Hypertension. Int. J. Pharm..

[94] Veeroju S., Kojonazarov B., Weiss A., Ghofrani H.A., Weissmann N., Grimminger F., Seeger W., Novoyatleva T., Schermuly R.T. (2021). Therapeutic Potential of Regorafenib—A Multikinase Inhibitor in Pulmonary Hypertension. Int. J. Mol. Sci..

[95] Li Z., Dong X., Wang Z., Liu W., Deng N., Ding Y., Tang L., Hla T., Zeng R., Li L. (2005). Regulation of PTEN by Rho small GTPases. Nature.

[96] Gao S.-Y., Li C.-Y., Chen J., Pan L., Saito S., Terashita T., Saito K., Miyawaki K., Shigemoto K., Mominoki K. (2004). Rho-ROCK Signal Pathway Regulates Microtubule-Based Process Formation of Cultured Podocytes—Inhibition of ROCK Promoted Process Elongation. Nephron Exp. Nephrol..

[97] Kosako H., Yoshida T., Matsumura F., Ishizaki T., Narumiya S., Inagaki M. (2000). Rho-kinase/ROCK is involved in cytokinesis through the phosphorylation of myosin light chain and not ezrin/radixin/moesin proteins at the cleavage furrow. Oncogene.

[98] Yasui Y., Amano M., Nagata K.-I., Inagaki N., Nakamura H., Saya H., Kaibuchi K., Inagaki M. (1998). Roles of Rho-associated Kinase in Cytokinesis; Mutations in Rho-associated Kinase Phosphorylation Sites Impair Cytokinetic Segregation of Glial Filaments. J. Cell Biol..

[99] Riento K., Ridley A.J. (2003). ROCKs: Multifunctional kinases in cell behaviour. Nat. Rev. Mol. Cell Biol..

[100] Guilluy C., Eddahibi S., Agard C., Guignabert C., Izikki M., Tu L., Savale L., Humbert M., Fadel E., Adnot S. (2009). RhoA and Rho Kinase Activation in Human Pulmonary Hypertension. Am. J. Respir. Crit. Care Med..

[101] Zhang Y., Wu S. (2017). Effects of fasudil on pulmonary hypertension in clinical practice. Pulm. Pharmacol. Ther..

[102] Otani N., Tomoe T., Kawabe A., Sugiyama T., Horie Y., Sugimura H., Yasu T., Nakamoto T. (2022). Recent Advances in the Treatment of Pulmonary Arterial Hypertension. Pharmaceuticals.

[103] Fagan K.A., Oka M., Bauer N.R., Gebb S.A., Ivy D.D., Morris K.G., McMurtry I.F. (2004). Attenuation of acute hypoxic pulmonary vasoconstriction and hypoxic pulmonary hypertension in mice by inhibition of Rho-kinase. Am. J. Physiol. Cell. Mol. Physiol..

[104] Dahal B.K., Kosanovic D., Pamarthi P.K., Sydykov A., Lai Y.-J., Kast R., Schirok H., Stasch J.-P., Ghofrani H.A., Weissmann N. (2010). Therapeutic efficacy of azaindole-1 in experimental pulmonary hypertension. Eur. Respir. J..

[105] Dhaliwal J.S., Badejo A.M., Casey D.B., Murthy S.N., Kadowitz P.J. (2009). Analysis of Pulmonary Vasodilator Responses to SB-772077-B [4-(7-((3-Amino-1-pyrrolidinyl)carbonyl)-1-ethyl-1H-imidazo(4,5-c)pyridin-2-yl)-1,2,5-oxadiazol-3-amine], a Novel Aminofurazan-Based Rho Kinase Inhibitor. Experiment.

[106] Fukumoto Y., Matoba T., Ito A., Takata-Tanaka H., Kishi T., Hayashidani S., Abe K., Takeshita A., Shimokawa H. (2005). Acute vasodilator effects of a Rho-kinase inhibitor, fasudil, in patients with severe pulmonary hypertension. Heart.

[107] Ishikura K., Yamada N., Ito M., Ota S., Nakamura M., Isaka N., Nakano T. (2006). Beneficial Acute Effects of Rho-Kinase Inhibitor in Patients With Pulmonary Arterial Hypertension. Circ. J..

[108] Fujita H., Fukumoto Y., Saji K., Sugimura K., Demachi J., Nawata J., Shimokawa H. (2010). Acute vasodilator effects of inhaled fasudil, a specific Rho-kinase inhibitor, in patients with pulmonary arterial hypertension. Heart Vessel..

[109] Jiang X., Wang Y.-F., Zhao Q.-H., Jiang R., Wu Y., Peng F.-H., Xu X.-Q., Wang L., He J., Jing Z.-C. (2014). Acute hemodynamic response of infused fasudil in patients with pulmonary arterial hypertension: A randomized, controlled, crossover study. Int. J. Cardiol..

[110] Fukumoto Y., Yamada N., Matsubara H., Mizoguchi M., Uchino K., Yao A., Kihara Y., Kawano M., Watanabe H., Takeda Y. (2013). Double-Blind, Placebo-Controlled Clinical Trial With a Rho-Kinase Inhibitor in Pulmonary Arterial Hypertension. Circ. J..

[111] Ranchoux B., Meloche J., Paulin R., Boucherat O., Provencher S., Bonnet S. (2016). DNA Damage and Pulmonary Hypertension. Int. J. Mol. Sci..

[112] Piao L., Sidhu V.K., Fang Y.-H., Ryan J.J., Parikh K.S., Hong Z., Toth P., Morrow E., Kutty S., Lopaschuk G.D. (2013). FOXO1-mediated upregulation of pyruvate dehydrogenase kinase-4 (PDK4) decreases glucose oxidation and impairs right ventricular function in pulmonary hypertension: Therapeutic benefits of dichloroacetate. J. Mol. Med..

[113] Michelakis E.D., McMurtry M.S., Wu X.-C., Dyck J.R., Moudgil R., Hopkins T.A., Lopaschuk G.D., Puttagunta L., Waite R., Archer S.L. (2002). Dichloroacetate, a Metabolic Modulator, Prevents and Reverses Chronic Hypoxic Pulmonary Hypertension in Rats. Circulation.

[114] Tian L., Wu D., Dasgupta A., Chen K.H., Mewburn J., Potus F., Lima P.D.A., Hong Z., Zhao Y.Y., Hindmarch C.C.T. (2020). Epigenetic Metabolic Reprogramming of Right Ventricular Fibroblasts in Pulmonary Arterial Hypertension. Circ. Res..

[115] Michelakis E.D., Gurtu V., Webster L., Barnes G., Watson G., Howard L., Cupitt J., Paterson I., Thompson R.B., Chow K. (2017). Inhibition of pyruvate dehydrogenase kinase improves pulmonary arterial hypertension in genetically susceptible patients. Sci. Transl. Med..

[116] Budas G.R., Boehm M., Kojonazarov B., Viswanathan G., Tian X., Veeroju S., Novoyatleva T., Grimminger F., Hinojosa-Kirschenbaum F., Ghofrani H.A. (2018). ASK1 Inhibition Halts Disease Progression in Preclinical Models of Pulmonary Arterial Hypertension. Am. J. Respir. Crit. Care Med..

[117] Boucherat O., Provencher S., Bonnet S. (2018). Therapeutic Value of ASK1 Inhibition in Pulmonary Arterial Hypertension. Am. J. Respir. Crit. Care Med..

[118] Rosenkranz S., Feldman J., McLaughlin V.V., Rischard F., Lange T.J., White R.J., Peacock A.J., Gerhardt F., Ebrahimi R., Brooks G. (2022). Selonsertib in adults with pulmonary arterial hypertension (ARROW): A randomised, double-blind, placebo-controlled, phase 2 trial. Lancet Respir. Med..

[119] Gong M., Fragakis N., Zhang C., Zhang Z., Li G., Liu T. (2016). Ranolazine as a novel therapy for pulmonary arterial hypertension. Int. J. Cardiol..

[120] Rocchetti M., Sala L., Rizzetto R., Staszewsky L.I., Alemanni M., Zambelli V., Russo I., Barile L., Cornaghi L., Altomare C. (2014). Ranolazine prevents INaL enhancement and blunts myocardial remodelling in a model of pulmonary hypertension. Cardiovasc. Res..

[121] Liles J.T., Hoyer K., Oliver J., Chi L., Dhalla A.K., Belardinelli L. (2015). Ranolazine Reduces Remodeling of the Right Ventricle and Provoked Arrhythmias in Rats with Pulmonary Hypertension. Experiment.

[122] Tocchetti C.G., Carpi A., Coppola C., Quintavalle C., Rea D., Campesan M., Arcari A., Piscopo G., Cipresso C., Monti M.G. (2014). Ranolazine protects from doxorubicin-induced oxidative stress and cardiac dysfunction. Eur. J. Heart Fail..

[123] Gomberg-Maitland M., Schilz R., Mediratta A., Addetia K., Coslet S., Thomeas V., Gillies H., Oudiz R.J. (2015). Phase I Safety Study of Ranolazine in Pulmonary Arterial Hypertension. Pulm. Circ..

[124] Khan S.S., Cuttica M.J., Beussink-Nelson L., Kozyleva A., Sanchez C., Mkrdichian H., Selvaraj S., Dematte J.E., Lee D.C., Shah S.J. (2015). Effects of Ranolazine on Exercise Capacity, Right Ventricular Indices, and Hemodynamic Characteristics in Pulmonary Arterial Hypertension: A Pilot Study. Pulm. Circ..

[125] Han Y., Forfia P.R., Vaidya A., Mazurek J.A., Park M.H., Ramani G., Chan S.Y., Waxman A.B. (2018). Rationale and design of the ranolazine PH–RV study: A multicentred randomised and placebo-controlled study of ranolazine to improve RV function in patients with non-group 2 pulmonary hypertension. Open Heart.

[126] Sutendra G., Bonnet S., Rochefort G., Haromy A., Folmes K.D., Lopaschuk G.D., Dyck J.R.B., Michelakis E.D. (2010). Fatty Acid Oxidation and Malonyl-CoA Decarboxylase in the Vascular Remodeling of Pulmonary Hypertension. Sci. Transl. Med..

[127] Leopold J.A., Maron B.A. (2016). Molecular Mechanisms of Pulmonary Vascular Remodeling in Pulmonary Arterial Hypertension. Int. J. Mol. Sci..

[128] Sharma S., Aldred M.A. (2020). DNA Damage and Repair in Pulmonary Arterial Hypertension. Genes.

[129] Xu D., Hu Y.-H., Gou X., Li F.-Y., Yang X.-Y., Li Y.-M., Chen F. (2022). Oxidative Stress and Antioxidative Therapy in Pulmonary Arterial Hypertension. Molecules.

[130] Xue C., Senchanthisai S., Sowden M., Pang J., White R.J., Berk B.C. (2020). Endothelial-to-Mesenchymal Transition and Inflammation Play Key Roles in Cyclophilin A–Induced Pulmonary Arterial Hypertension. Hypertension.

[131] Xu T., Shao L., Wang A., Liang R., Lin Y., Wang G., Zhao Y., Hu J., Liu S. (2020). *Retracted*: CD248 as a novel therapeutic target in pulmonary arterial hypertension. Clin. Transl. Med..

[132] Weise-Cross L., Resta T.C., Jernigan N.L. (2019). Redox Regulation of Ion Channels and Receptors in Pulmonary Hypertension. Antioxid. Redox Signal..

[133] Tang M., Wang R., Feng P., Dong Q., Chen W., Zhao Y., Li A., Li H., Chen J., Huang W. (2020). Dihydroartemisinin Attenuates Pulmonary Hypertension Through Inhibition of Pulmonary Vascular Remodeling in Rats. J. Cardiovasc. Pharmacol..

[134] Yu H., Liu J., Dong Y., Xu M., Xu L., Guan H., Xia X., Wang L. (2018). Anti-hypoxic effect of dihydroartemisinin on pulmonary artery endothelial cells. Biochem. Biophys. Res. Commun..

[135] Li Y., Cai H., Wei J., Zhu L., Yao Y., Xie M., Song L., Zhang C., Huang X., Wang L. (2022). Dihydroartemisinin Attenuates Hypoxic Pulmonary Hypertension via the Downregulation of miR-335 Targeting *Vangl2*. DNA Cell Biol..

[136] Suzuki Y., Yamaguchi K., Shimada S., Kitamura Y., Ohnishi H. (1982). Antithrombotic activity and the mechanism of action of trapidil (Rocornal^®^). Prostaglandins Leukot. Med..

[137] Türck P., Lacerda D.S., Carraro C.C., de Lima-Seolin B.G., Teixeira R.B., Bonetto J.H.P., Colombo R., Schenkel P.C., Belló-Klein A., Araujo A.S.D.R. (2018). Trapidil improves hemodynamic, echocardiographic and redox state parameters of right ventricle in monocrotaline-induced pulmonary arterial hypertension model. Biomed. Pharmacother..

[138] Zuckerbraun B.S., George P., Gladwin M.T. (2011). Nitrite in pulmonary arterial hypertension: Therapeutic avenues in the setting of dysregulated arginine/nitric oxide synthase signalling. Cardiovasc. Res..

[139] Simon M.A., Vanderpool R.R., Nouraie M., Bachman T.N., White P.M., Sugahara M., Gorcsan J., Parsley E.L., Gladwin M.T. (2016). Acute hemodynamic effects of inhaled sodium nitrite in pulmonary hypertension associated with heart failure with preserved ejection fraction. J. Clin. Investig..

[140] Sharp J., Farha S., Park M.M., Comhair S.A., Lundgrin E.L., Tang W.W., Bongard R.D., Merker M.P., Erzurum S.C. (2014). Coenzyme Q supplementation in pulmonary arterial hypertension. Redox Biol..

[141] Hoshikawa Y., Ono S., Suzuki S., Tanita T., Chida M., Song C., Noda M., Tabata T., Voelkel N.F., Fujimura S. (2001). Generation of oxidative stress contributes to the development of pulmonary hypertension induced by hypoxia. J. Appl. Physiol..

[142] Voelkel M.A., Wynne K.M., Badesch D.B., Groves B.M., Voelkel N.F. (2000). Hyperuricemia in Severe Pulmonary Hypertension. Chest.

[143] Bendayan D., Shitrit D., Ygla M., Huerta M., Fink G., Kramer M. (2003). Hyperuricemia as a prognostic factor in pulmonary arterial hypertension. Respir. Med..

[144] Spiekermann S., Schenk K., Hoeper M.M. (2009). Increased xanthine oxidase activity in idiopathic pulmonary arterial hypertension. Eur. Respir. J..

[145] Tan W., Madhavan K., Hunter K.S., Park D., Stenmark K.R. (2014). Vascular Stiffening in Pulmonary Hypertension: Cause or Consequence? (2013 Grover Conference Series). Pulm. Circ..

[146] Thenappan T., Chan S.Y., Weir E.K. (2018). Role of extracellular matrix in the pathogenesis of pulmonary arterial hypertension. Am. J. Physiol. Circ. Physiol..

[147] Poiani G.J., Tozzi C.A., Yohn S.E., Pierce R.A., Belsky S.A., Berg R.A., Yu S.Y., Deak S.B., Riley D.J. (1990). Collagen and elastin metabolism in hypertensive pulmonary arteries of rats. Circ. Res..

[148] Jones P.L., Rabinovitch M. (1996). Tenascin-C Is Induced With Progressive Pulmonary Vascular Disease in Rats and Is Functionally Related to Increased Smooth Muscle Cell Proliferation. Circ. Res..

[149] Kim Y.-M., Haghighat L., Spiekerkoetter E., Sawada H., Alvira C.M., Wang L., Acharya S., Rodriguez-Colon G., Orton A., Zhao M. (2011). Neutrophil Elastase Is Produced by Pulmonary Artery Smooth Muscle Cells and Is Linked to Neointimal Lesions. Am. J. Pathol..

[150] Thompson K., Rabinovitch M. (1996). Exogenous leukocyte and endogenous elastases can mediate mitogenic activity in pulmonary artery smooth muscle cells by release of extracellular matrix-bound basic fibroblast growth factor. J. Cell. Physiol..

[151] Bertero T., Cottrill K.A., Lu Y., Haeger C.M., Dieffenbach P., Annis S., Hale A., Bhat B., Kaimal V., Zhang Y.-Y. (2015). Matrix Remodeling Promotes Pulmonary Hypertension through Feedback Mechanoactivation of the YAP/TAZ-miR-130/301 Circuit. Cell Rep..

[152] Vieillard-Baron A., Frisdal E., Raffestin B., Baker A.H., Eddahibi S., Adnot S., D’Ortho M.-P. (2003). Inhibition of Matrix Metalloproteinases by Lung TIMP-1 Gene Transfer Limits Monocrotaline-Induced Pulmonary Vascular Remodeling in Rats. Hum. Gene Ther..

[153] Mawatari E., Hongo M., Sakai A., Terasawa F., Takahashi M., Yazaki Y., Kinoshita O., Ikeda U. (2007). Amlodipine prevents monocrotaline-induced pulmonary arterial hypertension and prolongs survival in rats independent of blood pressure lowering. Clin. Exp. Pharmacol. Physiol..

[154] Koo H.S., Kim K.C., Hong Y.M. (2011). Gene Expressions of Nitric Oxide Synthase and Matrix Metalloproteinase-2 in Monocrotaline-Induced Pulmonary Hypertension in Rats After Bosentan Treatment. Korean Circ. J..

[155] Peterson J.T. (2004). Matrix Metalloproteinase Inhibitor Development and the Remodeling of Drug Discovery. Heart Fail. Rev..

[156] Cowan K., Heilbut A., Humpl T., Lam C., Ito S., Rabinovitch M. (2000). Complete reversal of fatal pulmonary hypertension in rats by a serine elastase inhibitor. Nat. Med..

[157] Cowan K.N., Jones P.L., Rabinovitch M. (2000). Elastase and matrix metalloproteinase inhibitors induce regression, and tenascin-C antisense prevents progression, of vascular disease. J. Clin. Investig..

[158] Ilkiw R., Todorovich-Hunter L., Maruyama K., Shin J., Rabinovitch M. (1989). SC-39026, a serine elastase inhibitor, prevents muscularization of peripheral arteries, suggesting a mechanism of monocrotaline-induced pulmonary hypertension in rats. Circ. Res..

[159] Molteni A., Ward W.F., Ts’Ao C.-H., Hinz J.M. (1989). Monocrotaline-induced cardiopulmonary injury in rats. Biochem. Pharmacol..

[160] Zaidi S.H., You X.-M., Ciura S., Husain M., Rabinovitch M. (2002). Overexpression of the serine elastase inhibitor elafin protects transgenic mice from hypoxic pulmonary hypertension. Circulation.

[161] Nickel N.P., Spiekerkoetter E., Gu M., Li C.G., Li H., Kaschwich M., Diebold I., Hennigs J.K., Kim K.-Y., Miyagawa K. (2015). Elafin Reverses Pulmonary Hypertension via Caveolin-1–Dependent Bone Morphogenetic Protein Signaling. Am. J. Respir. Crit. Care Med..

[162] Sweatt A.J., Miyagawa K., Rhodes C.J., Taylor S., Del Rosario P.A., Hsi A., Haddad F., Spiekerkoetter E., Bental-Roof M., Bland R.D. (2021). Severe Pulmonary Arterial Hypertension Is Characterized by Increased Neutrophil Elastase and Relative Elafin Deficiency. Chest.

[163] West J., Niswender K.D., Johnson J.A., Pugh M.E., Gleaves L., Fessel J.P., Hemnes A.R. (2013). A potential role for insulin resistance in experimental pulmonary hypertension. Eur. Respir. J..

[164] Zamanian R.T., Hansmann G., Snook S., Lilienfeld D., Rappaport K.M., Reaven G.M., Rabinovitch M., Doyle R.L. (2008). Insulin resistance in pulmonary arterial hypertension. Eur. Respir. J..

[165] Davis B.J., Xie Z., Viollet B., Zou M.-H. (2006). Activation of the AMP-Activated Kinase by Antidiabetes Drug Metformin Stimulates Nitric Oxide Synthesis In Vivo by Promoting the Association of Heat Shock Protein 90 and Endothelial Nitric Oxide Synthase. Diabetes.

[166] Awad K.S., West J.D., Perez V., MacLean M. (2016). Novel Signaling Pathways in Pulmonary Arterial Hypertension (2015 Grover Conference Series). Pulm. Circ..

[167] Agard C., Rolli-Derkinderen M., Dumas-De-La-Roque E., Rio M., Sagan C., Savineau J., Loirand G., Pacaud P. (2009). Protective role of the antidiabetic drug metformin against chronic experimental pulmonary hypertension. Br. J. Pharmacol..

[168] Brittain E.L., Niswender K., Agrawal V., Chen X., Fan R., Pugh M.E., Rice T.W., Robbins I.M., Song H., Thompson C. (2020). Mechanistic Phase II Clinical Trial of Metformin in Pulmonary Arterial Hypertension. J. Am. Heart Assoc..

[169] Tyagi S., Gupta P., Saini A.S., Kaushal C., Sharma S. (2011). The peroxisome proliferator-activated receptor: A family of nuclear receptors role in various diseases. J. Adv. Pharm. Technol. Res..

[170] Sutliff R.L., Kang B.-Y., Hart C.M. (2010). PPARγ as a potential therapeutic target in pulmonary hypertension. Ther. Adv. Respir. Dis..

[171] Legchenko E., Chouvarine P., Borchert P., Fernandez-Gonzalez A., Snay E., Meier M., Maegel L., Mitsialis S.A., Rog-Zielinska E.A., Kourembanas S. (2018). PPARγ agonist pioglitazone reverses pulmonary hypertension and prevents right heart failure via fatty acid oxidation. Sci. Transl. Med..

[172] Lehman J.J., Boudina S., Banke N.H., Sambandam N., Han X., Young D.M., Leone T.C., Gross R.W., Lewandowski E.D., Abel E.D. (2008). The transcriptional coactivator PGC-1α is essential for maximal and efficient cardiac mitochondrial fatty acid oxidation and lipid homeostasis. Am. J. Physiol. Circ. Physiol..

[173] Zhang D., Wang G., Han D., Zhang Y., Xu J., Lu J., Li S., Xie X., Liu L., Dong L. (2014). Activation of PPAR-γ ameliorates pulmonary arterial hypertension via inducing heme oxygenase-1 and p21WAF1: An in vivo study in rats. Life Sci..

[174] Xie X., Wang G., Zhang D., Zhang Y., Zhu Y., Li F., Li S., Li M. (2015). Activation of peroxisome proliferator-activated receptor γ ameliorates monocrotaline-induced pulmonary arterial hypertension in rats. Biomed. Rep..

[175] Peters E.L., Bogaard H.J., Noordegraaf A.V., de Man F.S. (2021). Neurohormonal modulation in pulmonary arterial hypertension. Eur. Respir. J..

[176] Maron B.A., Leopold J.A., Hemnes A.R. (2020). Metabolic syndrome, neurohumoral modulation, and pulmonary arterial hypertension. Br. J. Pharmacol..

[177] Bristow M.R., Minobe W., Rasmussen R., Larrabee P., Skerl L., Klein J.W., Anderson F.L., Murray J., Mestroni L., Karwande S.V. (1992). Beta-adrenergic neuroeffector abnormalities in the failing human heart are produced by local rather than systemic mechanisms. J. Clin. Investig..

[178] Rain S., Andersen S., Najafi A., Schultz J.G., Bós D.D.S.G., Handoko M.L., Bogaard H.-J., Vonk-Noordegraaf A., Andersen A., van der Velden J. (2016). Right Ventricular Myocardial Stiffness in Experimental Pulmonary Arterial Hypertension. Circ. Heart Fail..

[179] Maron B.A., Leopold J.A. (2015). Emerging Concepts in the Molecular Basis of Pulmonary Arterial Hypertension. Circulation.

[180] García-Lunar I., Pereda D., Ibanez B., García-Álvarez A. (2020). Neurohormonal Modulation as a Therapeutic Target in Pulmonary Hypertension. Cells.

[181] Hemnes A.R., Rathinasabapathy A., Austin E.A., Brittain E.L., Carrier E.J., Chen X., Fessel J.P., Fike C.D., Fong P., Fortune N. (2018). A potential therapeutic role for angiotensin-converting enzyme 2 in human pulmonary arterial hypertension. Eur. Respir. J..

[182] Sandoval J., Del Valle-Mondragón L., Masso F., Zayas N., Pulido T., Teijeiro R., Gonzalez-Pacheco H., Olmedo-Ocampo R., Sisniega C., Paez-Arenas A. (2020). Angiotensin converting enzyme 2 and angiotensin (1–7) axis in pulmonary arterial hypertension. Eur. Respir. J..

[183] Perros F., Man F.H.-D., Bogaard H.J., Antigny F., Simonneau G., Bonnet S., Provencher S., Galiè N., Humbert M. (2017). Use of β-Blockers in Pulmonary Hypertension. Circ. Heart Fail..

[184] Badagliacca R., Mercurio V., Romeo E., Correale M., Masarone D., Papa S., Tocchetti C., Agostoni P. (2022). Beta-blockers in pulmonary arterial hypertension: Time for a second thought?. Vasc. Pharmacol..

[185] Bandyopadhyay D., Bajaj N.S., Zein J., Minai O.A., Dweik R.A. (2015). Outcomes of β-blocker use in pulmonary arterial hypertension: A propensity-matched analysis. Eur. Respir. J..

[186] Grinnan D., Bogaard H.-J., Grizzard J., Van Tassell B., Abbate A., DeWilde C., Priday A., Voelkel N.F. (2014). Treatment of Group I Pulmonary Arterial Hypertension with Carvedilol Is Safe. Am. J. Respir. Crit. Care Med..

[187] De Man F.S., Handoko M.L., van Ballegoij J.J., Schalij I., Bogaards S.J., Postmus P.E., van der Velden J., Westerhof N., Paulus W.J., Vonk-Noordegraaf A. (2012). Bisoprolol Delays Progression Towards Right Heart Failure in Experimental Pulmonary Hypertension. Circ. Heart Fail..

[188] Van Campen J.S., de Boer K., van de Veerdonk M.C., van der Bruggen C.E., Allaart C.P., Raijmakers P.G., Heymans M.W., Marcus J.T., Harms H.J., Handoko M.L. (2016). Bisoprolol in idiopathic pulmonary arterial hypertension: An explorative study. Eur. Respir. J..

[189] Simon M.A., Hanrott K., Budd D.C., Torres F., Grünig E., Escribano-Subias P., Meseguer M.L., Halank M., Opitz C., Hall D.A. (2022). An open-label, dose-escalation study to evaluate the safety, tolerability, pharmacokinetics, and pharmacodynamics of single doses of GSK2586881 in participants with pulmonary arterial hypertension. Pulm. Circ..

[190] Maron B.A., Leopold J.A. (2014). The Role of the Renin-Angiotensin-Aldosterone System in the Pathobiology of Pulmonary Arterial Hypertension (2013 Grover Conference Series). Pulm. Circ..

[191] Beckmann T., Shelley P., Patel D., Vorla M., Kalra D.K. (2022). Strategizing Drug Therapies in Pulmonary Hypertension for Improved Outcomes. Pharmaceuticals.

[192] Safdar Z., Cho E. (2021). Effect of spironolactone use in pulmonary arterial hypertension—Analysis from pivotal trial databases. Pulm. Circ..

[193] Favre J., Gao J., Di Zhang A., Remy-Jouet I., Ouvrard-Pascaud A., Dautreaux B., Escoubet B., Thuillez C., Jaisser F., Richard V. (2011). Coronary endothelial dysfunction after cardiomyocyte-specific mineralocorticoid receptor overexpression. Am. J. Physiol. Circ. Physiol..

[194] Preston I.R., Sagliani K.D., Warburton R.R., Hill N.S., Fanburg B.L., Jaffe I.Z., Penumatsa K.C., Toksoz D., Kharnaf M., Kapur N.K. (2013). Mineralocorticoid receptor antagonism attenuates experimental pulmonary hypertension. Am. J. Physiol. Cell. Mol. Physiol..

[195] Soderman C., Eriksson L.S., Juhlin-Dannfelt A., Lundberg J.M., Broman L., Holmgren A. (1993). Effect of vasoactive intestinal polypeptide (VIP) on pulmonary ventilation-perfusion relationships and central haemodynamics in healthy subjects. Clin. Physiol. Funct. Imaging.

[196] Busto R., Prieto J.C., Bodega G., Zapatero J., Carrero I. (2000). Immunohistochemical localization and distribution of VIP/PACAP receptors in human lung☆. Peptides.

[197] Petkov V., Mosgoeller W., Ziesche R., Raderer M., Stiebellehner L., Vonbank K., Funk G.-C., Hamilton G., Novotny C., Burian B. (2003). Vasoactive intestinal peptide as a new drug for treatment of primary pulmonary hypertension. J. Clin. Investig..

[198] Leuchte H.H., Baezner C., Baumgartner R.A., Bevec D., Bacher G., Neurohr C., Behr J. (2008). Inhalation of vasoactive intestinal peptide in pulmonary hypertension. Eur. Respir. J..

[199] Lazarus H.M., Denning J., Wring S., Palacios M., Hoffman S., Crizer K., Kamau-Kelley W., Symonds W., Feldman J. (2022). A trial design to maximize knowledge of the effects of rodatristat ethyl in the treatment of pulmonary arterial hypertension (ELEVATE 2). Pulm. Circ..

[200] Lazarus H., Denning J., Kamau-Kelley W., Wring S., Palacios M., Humbert M. (2021). ELEVATE 2: A multicenter study of rodatristat ethyl in patients with WHO Group 1 pulmonary arterial hypertension (PAH). Eur. Respir. J..

[201] Lahm T., Crisostomo P.R., Markel T.A., Wang M., Wang Y., Weil B., Meldrum D.R. (2008). Exogenous estrogen rapidly attenuates pulmonary artery vasoreactivity and acute hypoxic pulmonary vasoconstriction. Shock.

[202] Liu A., Philip J., Vinnakota K.C., Bergh F.V.D., Tabima D.M., Hacker T., Beard D.A., Chesler N.C. (2017). Estrogen maintains mitochondrial content and function in the right ventricle of rats with pulmonary hypertension. Physiol. Rep..

[203] Ventetuolo C.E., Baird G.L., Barr R.G., Bluemke D.A., Fritz J.S., Hill N.S., Klinger J.R., Lima J.A.C., Ouyang P., Palevsky H.I. (2016). Higher Estradiol and Lower Dehydroepiandrosterone-Sulfate Levels Are Associated with Pulmonary Arterial Hypertension in Men. Am. J. Respir. Crit. Care Med..

[204] Yang Y., Lin F., Xiao Z., Sun B., Wei Z., Liu B., Xue L., Xiong C. (2020). Investigational pharmacotherapy and immunotherapy of pulmonary arterial hypertension: An update. Biomed. Pharmacother..

[205] Kawut S.M., Archer-Chicko C.L., DeMichele A., Fritz J.S., Klinger J.R., Ky B., Palevsky H.I., Palmisciano A.J., Patel M., Pinder D. (2016). Anastrozole in Pulmonary Arterial Hypertension. A Randomized, Double-Blind, Placebo-controlled Trial. Am. J. Respir. Crit. Care Med..

[206] Chen X., Austin E.D., Talati M., Fessel J.P., Farber-Eger E.H., Brittain E.L., Hemnes A.R., Loyd J.E., West J. (2017). Oestrogen inhibition reverses pulmonary arterial hypertension and associated metabolic defects. Eur. Respir. J..

[207] De La Roque E.D., Savineau J.-P., Metivier A.-C., Billes M.-A., Kraemer J.-P., Doutreleau S., Jougon J., Marthan R., Moore N., Fayon M. (2012). Dehydroepiandrosterone (DHEA) improves pulmonary hypertension in chronic obstructive pulmonary disease (COPD): A pilot study. Ann. D’Endocrinol..

[208] Rhodes C.J., Howard L.S., Busbridge M., Ashby D., Kondili E., Gibbs J.S.R., Wharton J., Wilkins M.R. (2011). Iron Deficiency and Raised Hepcidin in Idiopathic Pulmonary Arterial Hypertension. J. Am. Coll. Cardiol..

[209] Smith T.G., Balanos G.M., Croft Q.P.P., Talbot N.P., Dorrington K.L., Ratcliffe P.J., Robbins P.A. (2008). The increase in pulmonary arterial pressure caused by hypoxia depends on iron status. J. Physiol..

[210] Ren X., Dorrington K.L., Maxwell P., Robbins P. (2000). Effects of desferrioxamine on serum erythropoietin and ventilatory sensitivity to hypoxia in humans. J. Appl. Physiol..

[211] Cotroneo E., Ashek A., Wang L., Wharton J., Dubois O., Bozorgi S., Busbridge M., Alavian K.N., Wilkins M.R., Zhao L. (2015). Iron Homeostasis and Pulmonary Hypertension. Circ. Res..

[212] Wunderer F., Traeger L., Sigurslid H.H., Meybohm P., Bloch D.B., Malhotra R. (2020). The role of hepcidin and iron homeostasis in atherosclerosis. Pharmacol. Res..

[213] Ramakrishnan L., Pedersen S.L., Toe Q.K., Quinlan G.J., Wort S.J. (2018). Pulmonary Arterial Hypertension: Iron Matters. Front. Physiol..

[214] Zou H.-X., Qiu B.-Q., Lai S.-Q., Zhou X.-L., Gong C.-W., Wang L.-J., Yuan M.-M., He A.-D., Liu J.-C., Huang H. (2021). Iron Metabolism and Idiopathic Pulmonary Arterial Hypertension: New Insights from Bioinformatic Analysis. BioMed Res. Int..

[215] Howard L.S.G.E., He J., Watson G.M.J., Huang L., Wharton J., Luo Q., Kiely D.G., Condliffe R., Pepke-Zaba J., Morrell N.W. (2021). Supplementation with Iron in Pulmonary Arterial Hypertension. Two Randomized Crossover Trials. Ann. Am. Thorac. Soc..

[216] Tuder R.M., Radisavljevic Z., Shroyer K.R., Polak J.M., Voelkel N.F. (1998). Monoclonal Endothelial Cells in Appetite Suppressant–associated Pulmonary Hypertension. Am. J. Respir. Crit. Care Med..

[217] Meloche J., Pflieger A., Vaillancourt M., Paulin R., Potus F., Zervopoulos S., Graydon C., Courboulin A., Breuils-Bonnet S., Tremblay È. (2014). Role for DNA Damage Signaling in Pulmonary Arterial Hypertension. Circulation.

[218] Paulin R., Meloche J., Jacob M.H., Bisserier M., Courboulin A., Bonnet S. (2011). Dehydroepiandrosterone inhibits the Src/STAT3 constitutive activation in pulmonary arterial hypertension. Am. J. Physiol. Circ. Physiol..

[219] Federici C., Drake K.M., Rigelsky C.M., McNelly L.N., Meade S.L., Comhair S.A.A., Erzurum S.C., Aldred M.A. (2015). Increased Mutagen Sensitivity and DNA Damage in Pulmonary Arterial Hypertension. Am. J. Respir. Crit. Care Med..

[220] Agarwal S., Perez V.A.D.J. (2021). In Defense of the Nucleus: NUDT1 and Oxidative DNA Damage in Pulmonary Arterial Hypertension. Am. J. Respir. Crit. Care Med..

[221] Feng W., Wang J., Yan X., Zhai C., Shi W., Wang Q., Zhang Q., Li M. (2019). Paclitaxel alleviates monocrotaline-induced pulmonary arterial hypertension via inhibition of FoxO1-mediated autophagy. Naunyn-Schmiedebergs Arch. Pharmacol..

[222] Zhao Y., Yang J., Liao W., Liu X., Zhang H., Wang S., Wang D., Feng J., Yu L., Zhu W.-G. (2010). Cytosolic FoxO1 is essential for the induction of autophagy and tumour suppressor activity. Nat. Cell Biol..

[223] Wang S., Xia P., Huang G., Zhu P., Liu J., Ye B., Du Y., Fan Z. (2016). FoxO1-mediated autophagy is required for NK cell development and innate immunity. Nat. Commun..

[224] Voorburg J.A., Cats V.M., Buis B., Bruschke A.V. (1988). Balloon Angioplasty in the Treatment of Pulmonary Hypertension Caused by Pulmonary Embolism. Chest.

[225] Feinstein J., Goldhaber S.Z., Lock J.E., Ferndandes S.M., Landzberg M.J. (2001). Balloon Pulmonary Angioplasty for Treatment of Chronic Thromboembolic Pulmonary Hypertension. Circulation.

[226] Jin Q., Zhao Z.-H., Luo Q., Zhao Q., Yan L., Zhang Y., Li X., Yang T., Zeng Q.-X., Xiong C.-M. (2020). Balloon pulmonary angioplasty for chronic thromboembolic pulmonary hypertension: State of the art. World J. Clin. Cases.

[227] Taniguchi Y., Miyagawa K., Nakayama K., Kinutani H., Shinke T., Okada K., Okita Y., Hirata K.-I., Emoto N. (2014). Balloon pulmonary angioplasty: An additional treatment option to improve the prognosis of patients with chronic thromboembolic pulmonary hypertension. Eurointervention.

[228] Brenot P., Jaïs X., Taniguchi Y., Alonso C.G., Gerardin B., Mussot S., Mercier O., Fabre D., Parent F., Jevnikar M. (2019). French experience of balloon pulmonary angioplasty for chronic thromboembolic pulmonary hypertension. Eur. Respir. J..

[229] Ogawa A., Satoh T., Fukuda T., Sugimura K., Fukumoto Y., Emoto N., Yamada N., Yao A., Ando M., Ogino H. (2017). Balloon Pulmonary Angioplasty for Chronic Thromboembolic Pulmonary Hypertension. Circ. Cardiovasc. Qual. Outcomes.

[230] Magon W., Stepniewski J., Jonas K., Waligora M., Podolec P., Kopec G. (2019). P4679Changes in systemic inflammation and endothelial dysfunction after balloon pulmonary angioplasty. Eur. Heart J..

[231] Cannon J.E., Su L., Kiely D.G., Page K., Toshner M., Swietlik E., Treacy C., Ponnaberanam A., Condliffe R., Sheares K. (2016). Dynamic Risk Stratification of Patient Long-Term Outcome After Pulmonary Endarterectomy. Circulation.

[232] Kawakami T., Ogawa A., Miyaji K., Mizoguchi H., Shimokawahara H., Naito T., Oka T., Yunoki K., Munemasa M., Matsubara H. (2016). Novel Angiographic Classification of Each Vascular Lesion in Chronic Thromboembolic Pulmonary Hypertension Based on Selective Angiogram and Results of Balloon Pulmonary Angioplasty. Circ. Cardiovasc. Interv..

[233] Martín M.V., Melón N.M., González-Trevilla A.A., Cebada F.S., Nieto S.H., Cruz-Utrilla A., Hinojosa W., López-Gude M.J., Charterina S.A., Ostolaza Y.R. (2022). Balloon pulmonary angioplasty can be an effective and safe therapeutic option in non-surgical elderly patients. Front. Cardiovasc. Med..

[234] Ciarka A., Doan V., Velez-Roa S., Naeije R., van de Borne P. (2010). Prognostic Significance of Sympathetic Nervous System Activation in Pulmonary Arterial Hypertension. Am. J. Respir. Crit. Care Med..

[235] Wensel R., Jilek C., Dorr M., Francis D.P., Stadler H., Lange T., Blumberg F., Opitz C., Pfeifer M., Ewert R. (2009). Impaired cardiac autonomic control relates to disease severity in pulmonary hypertension. Eur. Respir. J..

[236] Velez-Roa S., Ciarka A., Najem B., Vachiery J.-L., Naeije R., van de Borne P. (2004). Increased Sympathetic Nerve Activity in Pulmonary Artery Hypertension. Circulation.

[237] Perros F., Ranchoux B., Izikki M., Bentebbal S., Happé C., Antigny F., Jourdon P., Dorfmüller P., Lecerf F., Fadel E. (2015). Nebivolol for Improving Endothelial Dysfunction, Pulmonary Vascular Remodeling, and Right Heart Function in Pulmonary Hypertension. J. Am. Coll. Cardiol..

[238] Zhou L., Zhang J., Jiang X.-M., Xie D.-J., Wang J.-S., Li L., Li B., Wang Z.-M., Rothman A.M., Lawrie A. (2015). Pulmonary Artery Denervation Attenuates Pulmonary Arterial Remodeling in Dogs With Pulmonary Arterial Hypertension Induced by Dehydrogenized Monocrotaline. JACC Cardiovasc. Interv..

[239] Chen S.-L., Zhang Y.-J., Zhou L., Xie D.-J., Zhang F.-F., Du-Jiang X., Wong S.S., Kwan T.W. (2013). Percutaneous pulmonary artery denervation completely abolishes experimental pulmonary arterial hypertension in vivo. Eurointervention.

[240] Rothman A.M., Arnold N.D., Chang W., Watson O., Swift A.J., Condliffe R., Elliot C.A., Kiely D.G., Suvarna S.K., Gunn J. (2015). Pulmonary Artery Denervation Reduces Pulmonary Artery Pressure and Induces Histological Changes in an Acute Porcine Model of Pulmonary Hypertension. Circ. Cardiovasc. Interv..

[241] Chen S.-L., Zhang F.-F., Xu J., Xie D.-J., Zhou L., Nguyen T., Stone G.W. (2013). Pulmonary Artery Denervation to Treat Pulmonary Arterial Hypertension. J. Am. Coll. Cardiol..

[242] Zhang H., Zhang J., Chen M., Xie D.-J., Kan J., Yu W., Li X.-B., Xu T., Gu Y., Dong J. (2019). Pulmonary Artery Denervation Significantly Increases 6-Min Walk Distance for Patients with Combined Pre- and Post-Capillary Pulmonary Hypertension Associated with Left Heart Failure. JACC Cardiovasc. Interv..

[243] Rubin L.J. (2015). Pulmonary Artery Denervation for Pulmonary Artery Hypertension. JACC Cardiovasc. Interv..

[244] Zhang H., Wei Y., Zhang C., Yang Z., Kan J., Gu H., Fan F., Gu H., Wang Q., Xie D. (2022). Pulmonary Artery Denervation for Pulmonary Arterial Hypertension: A Sham-Controlled Randomized Trial. JACC Cardiovasc. Interv..

[245] Kurzyna M., Dąbrowski M., Bielecki D., Fijalkowska A., Pruszczyk P., Opolski G., Burakowski J., Florczyk M., Tomkowski W.Z., Wawrzyńska L. (2007). Atrial Septostomy in Treatment of End-Stage Right Heart Failure in Patients with Pulmonary Hypertension. Chest.

[246] Rich S., Lam W. (1983). Atrial septostomy as palliative therapy for refractory primary pulmonary hypertension. Am. J. Cardiol..

[247] Sandoval J., Gaspar J., Pena H., Santos L.E., Cordova J., Del Valle K., Rodriguez A., Pulido T. (2011). Effect of atrial septostomy on the survival of patients with severe pulmonary arterial hypertension. Eur. Respir. J..

[248] Yan C., Wan L., Li H., Wang C., Guo T., Niu H., Li S., Yundan P., Wang L., Fang W. (2022). First in-human modified atrial septostomy combining radiofrequency ablation and balloon dilation. Heart.

[249] Dai Y., Chen X., Song X., Chen X., Ma W., Lin J., Wu H., Hu X., Zhou Y., Zhang H. (2019). Immunotherapy of Endothelin-1 Receptor Type A for Pulmonary Arterial Hypertension. J. Am. Coll. Cardiol..

[250] Bockmeyer C.L., Maegel L., Janciauskiene S., Rische J., Lehmann U., Maus U.A., Nickel N., Haverich A., Hoeper M., Golpon H.A. (2012). Plexiform vasculopathy of severe pulmonary arterial hypertension and microRNA expression. J. Heart Lung Transplant..

[251] Reynolds A.M., Xia W., Holmes M.D., Hodge S.J., Danilov S., Curiel D.T., Morrell N.W., Reynolds P.N. (2007). Bone morphogenetic protein type 2 receptor gene therapy attenuates hypoxic pulmonary hypertension. Am. J. Physiol. Cell. Mol. Physiol..

